# A phase II, single-center, double-blind, randomized placebo-controlled trial to explore the efficacy and safety of intravenous melatonin in patients with COVID-19 admitted to the intensive care unit (MelCOVID study): a structured summary of a study protocol for a randomized controlled trial

**DOI:** 10.1186/s13063-020-04632-4

**Published:** 2020-08-05

**Authors:** Miguel Rodríguez-Rubio, Juan Carlos Figueira, Darío Acuña-Castroviejo, Alberto M. Borobia, Germaine Escames, Pedro de la Oliva

**Affiliations:** 1 Pediatric Intensive Care Department, Hospital Universitario La Paz, Madrid, Spain; 2 Department of Pediatrics, School of Medicine, Universidad Autónoma de Madrid, Madrid, Spain; 3 Intensive Care Medicine Department, Hospital Universitario La Paz, Madrid, Spain; 4 Department of Physiology, Faculty of Medicine, Biomedical Research Center, Health Science Technology Park, University of Granada, Granada, Spain; 5 CIBERfes, Ibs Granada, Granada, Spain; 6 Clinical Laboratories, San Cecilio University Hospital, Granada, Spain; 7 Clinical Pharmacology Department, Clinical Trial Unit, Hospital Universitario La Paz – IdiPAZ, Madrid, Spain; 8 School of Medicine, Universidad Autónoma de Madrid, Madrid, Spain

**Keywords:** COVID-19, Randomized Controlled Trial, protocol, melatonin, treatment, intensive care, ARDS, inflammation

## Abstract

**Objectives:**

• Primary objective: to evaluate the effect of intravenous melatonin (IVM) on mortality in adult patients admitted to the intensive care unit (ICU) with COVID-19.

• Secondary objectives:

◦ To evaluate the effect of IVM on ICU length of stay.

◦ To evaluate the effect of IVM on the length of mechanical ventilation (MV).

◦ To evaluate if the use of IVM is associated with an increase in the number of ventilator-free days.

◦ To evaluate if the use of IVM is associated with a reduced number of failing organs as determined by the sequential organ failure assessment (SOFA) scale.

◦ To evaluate if the use of IVM is associated with a reduction of the frequency and severity of COVID-19-associated thromboembolic phenomena.

◦ To evaluate if the use of IVM is associated with a decreased systemic inflammatory response assessed by plasma levels of ferritin, D-dimer, C-reactive protein, procalcitonin and interleukin-6.

◦ To evaluate if the use of IVM is associated with an improvement in hematologic parameters.

◦ To evaluate if the use of IVM is associated with an improvement in biochemical parameters.

◦ To evaluate if the use of IVM is associated with an improvement in blood gas analysis parameters.

◦ To evaluate adverse events during the 28 day study period.

**Trial design:**

Phase II, single center, double-blind, placebo-controlled randomized trial with a two-arm parallel group design and 2:1 allocation ratio.

**Participants:**

Only critically ill adult patients that fulfill all of the inclusion criteria and none of the exclusion criteria will be included. The study will be conducted in a mixed ICU of a publicly funded tertiary referral center in Madrid, Spain with a 30-bed capacity and 1100 admissions per year.

• Inclusion criteria:

◦ Patient, family member or legal guardian has provided written Informed Consent.

◦ Age ε 18 years.

◦ Confirmed SARS-CoV-2 infection with compatible symptoms AND a positive RT-PCR.

◦ Admission to the ICU with acute hypoxemic respiratory failure attributed to SARS-CoV-2 infection.

◦ ICU length of stay of less than 7 days prior to randomization with or without MV and without signs of improvement in respiratory failure (MURRAY score at randomization greater or equal to the MURRAY score at ICU admission).

• Exclusion criteria:

◦ Participant in a different COVID-19 study in which the study drug is under clinical development and hasn’t been previously authorized for commercialization.

◦ Liver enzymes > 5 times the upper normal range.

◦ Chronic kidney disease with GFR < 30 mL/min/1.73 m^2^ (stage 4 or greater) or need for hemodialysis.

◦ Pregnancy. A pregnancy test will be performed on every woman younger than 55 years of age prior to inclusion.

◦ Terminal surgical or medical illness.

◦ Autoimmune disease.

◦ Any patient condition that can prevent the study procedures to be carried out at the treating physician’s judgement.

**Intervention and comparator:**

All patients will receive standard-of-care treatment according to the current institutional protocols. In addition, patients will be randomized in a 2:1 ratio to receive:

• Experimental group (12 patients): 7 days of 5 mg per Kg of actual body weight per day of intravenous melatonin every 6 hours. Maximum daily dose 500 mg per day.

• Control group (6 patients): 7 days of 5 mg per Kg of actual body weight per day of intravenous identically-looking placebo every 6 hours.

After 3 days of treatment, 3 intensive care physicians will evaluate the participant and decide whether or not to complete the treatment based on their clinical assessment:

• If objective or subjective signs of improvement or no worsening of the general clinical condition, respiratory failure, inflammatory state or multi-organ failure are observed, the participant will continue the treatment until completion.

• If an adverse effect or clinical impairment is observed that is objectively or subjectively attributable to the study drug the treatment will be stopped.

**Main outcome:**

Mortality in each study group represented in frequency and time-to-event at day 28 after randomization

**Randomization:**

The randomization sequence was created using SAS version 9.4 statistical software (programmed and validated macros) with a 2:1 allocation. No randomization seed was pre-specified. The randomization seed was generated using the time on the computer where the program was executed.

**Blinding (masking):**

Participants, caregivers and study groups will be blinded to arm allocation.

**Numbers to be randomized (sample size):**

A total of 18 patients will be randomized in this trial: 12 to the experimental arm and 6 to the control arm.

**Trial Status:**

Protocol version 2.0, June 5^th^ 2020.

Trial status: recruitment not started. The first patient is expected to be recruited in October 2020. The last patient is anticipated to be recruited in August 2021.

**Trial registration:**

EU Clinical Trials Register. Date of trial registration: 10 July 2020. URL: https://www.clinicaltrialsregister.eu/ctr-search/trial/2020-001808-42/ES

**Full protocol:**

The full protocol is attached as an additional file, accessible from the Trials website (Additional file 1). In the interest of expediting dissemination of this material, the familiar formatting has been eliminated; this Letter serves as a summary of the key elements of the full protocol.

## Supplementary information

**Additional file 1.** Full Study Protocol.

## Data Availability

Not applicable

