# Supplementary material for: A phase II, single-center, double-blind, randomized placebo-controlled trial to explore the efficacy and safety of intravenous melatonin in patients with COVID-19 admitted to the intensive care unit (MelCOVID study): a structured summary of a study protocol for a randomized controlled trial
Source: Trials. 2020 Aug 5;21:699. doi: 10.1186/s13063-020-04632-4 (PMC7403786; doi:10.1186/s13063-020-04632-4)
Supplement: Supplementary file 1 — Additional file 1. Full Study Protocol. [file 13063_2020_4632_MOESM1_ESM.pdf]

ENSAYO CLÍNICO DE FASE II, UNICÉNTRICO, DOBLE CIEGO, ALEATORIZADO, CONTROLADO CON PLACEBO PARA EXPLORAR LA EFICACIA Y SEGURIDAD DE MELATONINA I.V. EN PACIENTES CON COVID-19 INGRESADOS EN UCI (ESTUDIO MELCOVID)

Código Protocolo: PHM-2020-001

Nº EudraCT: 2020-001808-42

Fármaco del estudio: melatonina

Fase del Estudio: Fase II

Versión: v2.0

Fecha: 05-junio-2020

Confidencial

Este protocolo contiene información privilegiada o confidencial que es propiedad del Promotor. La información no puede ser revelada a un tercero sin la autorización del Promotor.

DATOS ADMINISTRATIVOS

Promotor

PHARMAMEL S.L.

Domicilio de gestión: Centro de Transferencia Tecnológica, Gran Vía nº 48, 7º Planta, 18071 Granada.
Domicilio social: C/ Mulhacén nº 40, CD31, 18152 Dílar (Granada).

Personas de contacto:
Germaine Escames
Darío Acuña Castroviejo
Email:
gescames@ugr.es
dacuna@ugr.es
Teléfonos:
618521646
616929320

Organización de investigación por contrato (CRO)

Nombre de la empresa: TFS

Medicon Village, Scheelevägen 2, Lund, Suecia
Teléfono/Fax: +46 462 801 800/+46 462 801 801

Project Manager de la CRO: Maria del Sol Díaz Infantes
Avenida de Burgos, 12B, 4º IZDA, 28036 MADRID
Email: Marisol.Diaz@tfscro.com
Teléfono: +34 670 05 50 14

Investigador Principal

Dr. D. Juan Carlos Figueira
Servicio de Medicina Intensiva.
Hospital Universitario La Paz
Paseo de la Castellana, 261. 28046 Madrid
Teléfono: 91 727 7124 /7149 /7358
FAX: 91 727 7358

Comité Científico

Servicio de Farmacología Clínica

Dr. D. Alberto Borobia
Dra. Dª. Irene García García

Servicio de Cuidados Intensivos Pediátricos

Dr. D. Pedro de la Oliva
Dr. D. Miguel Rodríguez Rubio

Servicio de Medicina Intensiva

Dr. D. Juan Carlos Figueira
Dr. D. José Manuel Añón

Servicio de Medicina Interna. Enfermedades Infecciosas

Dr. José Ramón Arribas

Servicio de Microbiología

Dr. Julio García Rodríguez

Departamento de Fisiología. Universidad de Granada

Prof. D. Darío Acuña
Prof. Dña. Germaine Escames

Centros participantes

Hospital Universitario La Paz

Comité de Ética de la Investigación que evalúa el estudio

- CEIm del Hospital Universitario La Paz (Madrid)

RESUMEN

i. TÍTULO

Ensayo clínico de fase II, unicéntrico, doble ciego, aleatorizado, controlado con placebo para explorar la eficacia y seguridad de **MEL**atonina i.v. en pacientes con **COVID**-19 ingresados en UCI (**estudio MELCOVID**).

ii. FASE DEL ESTUDIO

Ensayo clínico fase II.

iii. MEDICAMENTO EN INVESTIGACIÓN

Melatonina 6 mg/mL solución inyectable y para perfusión.

iv. ENFERMEDAD A ESTUDIO

Infección por SARS-CoV-2 (COVID-19).

v. SUJETOS PARTICIPANTES

Pacientes adultos diagnosticados de COVID-19 que presenten criterios de ingreso en UCI.

vi. NÚMERO DE PACIENTES

18 pacientes (12 pacientes recibirán el tratamiento experimental y 6 pacientes recibirán placebo).

vii. CENTRO PARTICIPANTE

SERVICIO	CENTRO
Unidad de Cuidados Intensivos (Servicio de Medicina Intensiva)	Hospital Universitario La Paz (Madrid)

viii. CRITERIOS DE SELECCIÓN

En el estudio se incluirán los pacientes que cumplan TODOS los criterios de inclusión y NINGUNO de los criterios de exclusión.

Criterios de inclusión:

- Otorgamiento de consentimiento informado por parte del paciente, su familiar o su representante legal.
- Edad igual o superior a 18 años.
- Presencia de infección por SARS-CoV-2, con clínica compatible y confirmación mediante PCR.
- Ingreso en UCI con insuficiencia respiratoria aguda hipoxémica atribuidos a la infección por SARS-CoV-2
- Llevar ingresado en UCI menos de 7 dias, con o sin ventilación mecánica (VM) y sin signos de mejoría de la insuficiencia respiratoria (puntuación de MURRAY igual o superior a la puntuación presentada al ingreso en UCI).

Criterios de exclusión:

- Estar incluido en otro estudio de COVID-19 que implique la administración de un medicamento en fase de desarrollo clínico que carezca de una autorización de comercialización previa.
- Transaminasas hepáticas > 5 veces el LSN.
- Insuficiencia renal de estadio IV o en diálisis (GFR <30)
- Embarazo. Se realizará un test de embarazo en toda mujer elegible menor 55 años antes de su inclusión.
- Enfermedad terminal médica o quirúrgica.
- Enfermedad autoinmune.
- Cualquier condición del paciente que en opinión del investigador impida llevar a cabo los procedimientos del estudio.

ix. OBJETIVOS

Objetivo principal:

- Evaluar si el tratamiento con melatonina i.v. reduce la mortalidad en pacientes con COVID-19 ingresados en UCI.

Objetivos secundarios:

- Evaluar si el tratamiento con melatonina i.v. reduce la duración del ingreso en la UCI.
- Evaluar si el tratamiento con melatonina i.v. reduce la duración de la ventilación mecánica (VM).
- Evaluar si el tratamiento con melatonina i.v. se asocia a un aumento de días libres de VM.
- Evaluar si el tratamiento con melatonina i.v. se asocia a una reducción del número de fallos de órganos vitales, según la escala SOFA.
- Evaluar si el tratamiento con melatonina i.v. se asocia a una reducción de frecuencia y gravedad de los procesos trombo-embólicos causados por COVID-19.

- Evaluar si el tratamiento con melatonina i.v. se asocia a una reducción de la respuesta inflamatoria sistémica, definida por los niveles de ferritina, dímero-D, proteína C reactiva (PCR) o procalcitonina (PCT) e IL-6.
- Evaluar si el tratamiento con melatonina i.v. se asocia a mejora de parámetros hematológicos.
- Evaluar si el tratamiento con melatonina i.v. se asocia a mejora de parámetros bioquímicos.
- Evaluar si el tratamiento con melatonina i.v. se asocia a mejora de parámetros gasométricos.
- Describir los acontecimientos y reacciones adversas acontecidos durante el estudio.

x. GRUPOS DE TRATAMIENTO

Todos los pacientes recibirán el tratamiento *standard-of-care* (SOC) definido por el protocolo vigente del centro en el momento del inicio del estudio, y serán aleatorizados en una proporción 2:1 a recibir, además:

- **grupo experimental** (12 pacientes): melatonina por vía i.v. con el siguiente esquema de dosis dependiendo del peso corporal: 5 mg/kg peso actual/día repartidos en 4 dosis al día (1 dosis/6h) y con una dosis máxima diaria de 500 mg.
- **grupo control** (6 pacientes): placebo por vía i.v.: 5 mg/kg/día repartidos en 4 dosis al día (1 dosis/6h).

xi. CRITERIOS DE EVALUACIÓN

Evaluación principal: mortalidad en cada grupo de tratamiento, en frecuencias y en tiempo hasta el evento.

Evaluaciones secundarias de eficacia: tiempo de ingreso en UCI, tiempo con ventilación mecánica y modificación del estado de gravedad del paciente respecto a los valores basales:

- Manifestaciones clínicas.
- Escala SOFA.
- Escala de Glasgow.
- Escala APACHE II.
- Escala ordinal de mejoría clínica de la OMS.
- Parámetros inflamatorios:
    - PCR, PCT, troponina, Dímero-D, IL-6, ferritina.
- Parámetros de coagulación:
    - Plaquetas, fibrinógeno, Tº Cefalina, Tº Protrombina, Antitrombina III, ADAMTS13 (Ac Anti ADAMTS13 si ADAMTS < 20%), Factor Xa.
- Parámetros bioquímicos:
    - CK, LDH, GOT, GPT, bilirrubina, Vit D y 1,25-OH-Vit D (calcitriol), calcio, albumina, BUN, creatinina, mioglobina.
- Parámetros hematológicos:
    - Hemograma, Hb libre
- Parámetros gasométricos arteriales y electrolitos:

    - pH, SaO_2, $PaCO_2$, PO_2, HCO_3, glucosa, Na, K, Cloro, Ca iónico, Hb, lactato, anión gap, PaO_2/FiO_2.
- Escala MURRAY.

Evaluaciones de seguridad: se recogerán a lo largo de todo el estudio:

- Acontecimientos adversos emergentes.
- Acontecimientos adversos emergentes relacionados con el tratamiento.
- Acontecimientos adversos emergentes por graduación de intensidad.
- Acontecimientos adversos emergentes graves (relacionados y no relacionados con el tratamiento).
- Acontecimientos adversos emergentes que llevan a la interrupción del estudio.
- Acontecimientos adversos emergentes con resultado de muerte.

xii. PERIODOS DEL ESTUDIO

Periodo de inclusión: 1 mes.

Periodo de tratamiento: 7 días

Periodo de evaluación: hasta 28 días

Periodo de seguimiento tras fin de tratamiento: 30 días.

Periodo de seguimiento tras fin de estudio: hasta el alta en UCI/Hospital

ÍNDICE

DATOS ADMINISTRATIVOS **2**

RESUMEN **4**

ABREVIATURAS **10**

1. ANTECEDENTES Y JUSTIFICACIÓN **12**
- 1.1 MELATONINA COMO TRATAMIENTO POTENCIAL EFICAZ FRENTE AL COVID 19 12
- 1.2 MEDICAMENTO EN INVESTIGACIÓN: MELATONINA 15
  - *1.2.1 Datos disponibles de eficacia y seguridad 15*
  - *1.2.2 Justificación de la selección de la dosis 16*
  - *1.2.3 Resumen de los riesgos y beneficios conocidos para los seres humanos 17*
- 1.3 JUSTIFICACIÓN DEL ESTUDIO 17

2. OBJETIVOS DEL ESTUDIO **19**
- 2.1 OBJETIVO PRINCIPAL 19
- 2.2 OBJETIVOS SECUNDARIOS 19

3. DISEÑO DEL ESTUDIO Y EVALUACIONES **19**
- 3.1 TIPO DE ESTUDIO 19
- 3.2 DISEÑO Y TRATAMIENTO DEL ESTUDIO 19
  - *3.2.1 Periodo de selección 19*
  - *3.2.2 Periodo de tratamiento 20*
  - *3.2.3 Periodo de seguimiento 20*
  - *3.2.4 Tratamiento del estudio 20*
  - *3.2.5 Asignación al grupo de tratamiento 20*
- 3.3 TERAPIAS CONCOMITANTES PROHIBIDAS O RESTRINGIDAS 21
  - *3.3.1 Terapias prohibidas 21*
  - *3.3.2 Terapias restringidas y precauciones 21*
- 3.4 VARIABLES DEL ESTUDIO 21
  - *3.4.1 Variable principal 21*
  - *3.4.2 Variables secundarias 21*

4. SELECCIÓN DE PACIENTES **22**
- 4.1 POBLACIÓN DEL ESTUDIO 22
- 4.2 CRITERIOS DE INCLUSIÓN 22
- 4.3 CRITERIOS DE EXCLUSIÓN 23

5. DETALLE DE LOS PROCEDIMIENTOS DEL ESTUDIO **23**
- 5.1 EVALUACIONES DE EFICACIA Y SEGURIDAD 23
- 5.2 PRUEBAS ANALÍTICAS 25
- 5.3 ESQUEMA TEMPORAL DE LOS PROCEDIMIENTOS 26
- 5.4 FIN DEL ESTUDIO Y PERÍODO DE SEGUIMIENTO 31
  - *5.4.1 Fin del estudio 31*
  - *5.4.2 Duración del seguimiento tras fin de estudio 31*

6. MEDICACIÓN DEL ESTUDIO **32**
- 6.1 IDENTIFICACIÓN DE LA MEDICACIÓN DEL ESTUDIO 32

6.2 SUMINISTRO 32
6.3 ENVASADO Y ETIQUETADO 32
6.4 MANEJO Y DISPENSACIÓN DE LA MEDICACIÓN DEL ESTUDIO 33
6.5 ADMINISTRACIÓN DE LA MEDICACIÓN DEL ESTUDIO 33
6.6 ERRORES DE MEDICACIÓN Y SOBREDOSIS 33
6.7 REGISTROS DE LA MEDICACIÓN DEL ESTUDIO EN LOS CENTROS INVESTIGADORES 33
6.8 DEVOLUCIÓN Y DESTRUCCIÓN DE LA MEDICACIÓN DEL ESTUDIO 34
6.8.1 Devolución de la medicación 34

7. SEGURIDAD Y NOTIFICACIÓN DE ACONTECIMIENTOS ADVERSOS 34
7.1 DEFINICIONES 34
7.2 RELACIÓN DE CAUSALIDAD ENTRE EL ACONTECIMIENTO ADVERSO Y EL MEDICAMENTO DEL ESTUDIO 35
7.3 EVALUACIÓN DE LA INTENSIDAD DE UN ACONTECIMIENTO ADVERSO 36
7.4 RECOPILACIÓN DE LA INFORMACIÓN SOBRE ACONTECIMIENTOS Y REACCIONES ADVERSAS 36
7.5 PROCEDIMIENTO PARA LA COMUNICACIÓN DE AAG 37
7.6 NOTIFICACIÓN EXPEDITIVA DE RAGI 37
7.7 SEGUIMIENTO DE LOS AA 37
7.8 ANOMALÍAS ANALÍTICAS 37
7.9 EMBARAZO 38
7.10 SOBREDOSIS Y ERRORES DE MEDICACIÓN 38
7.11 RESPONSABLE DE LA NOTIFICACIÓN DE AAG 38

8. METODOLOGÍA ESTADÍSTICA 39
8.1 DETERMINACIÓN DEL TAMAÑO DE LA MUESTRA 39
8.2 ANÁLISIS ESTADÍSTICO 39

9. ASPECTOS ÉTICOS Y NORMATIVOS 40
9.1 CUMPLIMIENTO NORMATIVO 40
9.2 RESPONSABILIDAD CIVIL 40
9.3 CONSENTIMIENTO INFORMADO 40
9.4 PROTECCIÓN DE DATOS 41

10. GESTIÓN DEL ESTUDIO 42
10.1 RETIRADA DE LOS PACIENTES DEL ESTUDIO 42
10.2 DESENMASCARAMIENTO Y APERTURA DEL CÓDIGO 42
10.3 MODIFICACIONES DEL PROTOCOLO 42

11. GESTION Y CALIDAD LOS DE DATOS 43
11.1 MONITORIZACIÓN 43
11.2 ARCHIVOS E INFORMES 43
11.3 RETENCIÓN DE ARCHIVOS 43
11.4 INSPECCIONES Y AUDITORÍAS 44

12. DIFUSIÓN DE RESULTADOS 44

13. INFORME FINAL DEL ESTUDIO 44

14. BIBLIOGRAFÍA 45

15. HOJA DE FIRMAS 51

ABREVIATURAS

AA	Acontecimiento adverso
AAG	Acontecimiento adverso grave
ACE2	Enzima convertidora de angiotensina II
ADN	Ácido desoxirribonucleico
AEMPS	Agencia Española de Medicamentos y Productos Sanitarios
APACHE	Acute Physiology And Chronic Health Evaluation
ARN	Ácido ribonucleico
CDC	Centro para el control de Enfermedades
CEIm	Comités de Ética de la Investigación con medicamentos
CID	Coagulación intravascular diseminada
CK	Creatin-kinasa
CoV	Coronavirus
COVID-19	Enfermedad infecciosa por coronavirus-19
CRD	Cuaderno de recogida de datos
ECMO	Oxigenación por membrana extracorpórea
GOT	Glutamato-oxalacetato transaminasa
GPT	Glutamato piruvato transaminasa
Hb	Hemoglobina
HTA	Hipertensión arterial
IFN-γ	Interferon gamma
IC95%	Intervalo de confianza del 95%
Ig	Immunoglobulina

IL	Interleucina
LDH	Lactatodeshidrogenasa
MedDRA	*Medical Dictionary for Regulatory Activities*
MERS	Síndrome respiratorio de Oriente Medio
NF-κB	*Factor nuclear potenciador de las cadenas ligeras kappa de las células B activadas*
OMS	Organización Mundial de la Salud
PRC	Reacción en cadena de la polimerasa
PCT	Procalcitonina
RA	Reacción adversa
RAG	Reacción adversa grave
RAGI	Reacción adversa grave e inesperada
RAI	Reacción adversa inesperada
ROS	Radicales libres
SARS	Síndrome respiratorio agudo grave
SDRA	Síndrome de distrés respiratorio agudo
SOC	*Standard-of-care*
SOFA	Sequential Organ Failure Assessment
TAC	Tomografía axial computarizada
TNF-α	Factor de necrosis tumoral alfa
UCI	Unidad de Cuidados Intensivos
VM	Ventilación mecánica

1. ANTECEDENTES Y JUSTIFICACIÓN

1.1 MELATONINA COMO TRATAMIENTO POTENCIAL EFICAZ FRENTE AL COVID 19

El COVID-19 es la enfermedad causada por el virus SARS-CoV-2, un β-coronavirus de tipo ARN, que previsiblemente proviene del murciélago, como así lo indican los estudios del genoma de COVID-19 y el CoV RaTG13 del murciélago. La transmisión humano-humano ocurre principalmente entre miembros familiares y amigos que pueden contactar con pacientes o portadores sanos. Los datos epidemiológicos a fecha de 8 de abril de 2020 indican un total de 1.434.426 infectados en el mundo, con 82.220 muertes y 301.768 pacientes recuperados, aunque estas cifran cambian diariamente. En España, al día de hoy hay 146.690 infectados, 14.555 fallecidos y 48.021 recuperados. Estas cifras dan idea de la severidad de esta pandemia.

El enzima convertidor de angiotensina II (ACE2), localizado en el tracto respiratorio de humanos, es el receptor para el SARS-CoV-2 [1]. La función de ACE2 es transformar la angiotensina I en angiotensina 1-9 y la angiotensina II en angiotensina 1-7. Estos productos tienen efectos vasodilatadores, antifibróticos, antiinflamatorios y favorecen la natriuresis. Son efectos que reducen la tensión arterial, contrarregulando la acción de la angiotensina II. La ACE2 se ha relacionado con la protección frente a la hipertensión arterial (HTA), la arteriosclerosis y otros procesos vasculares y pulmonares. Por el contrario, la enzima convertidora de la angiotensina (ACE), que transforma la Angiotensina I en Angiotensina II, favorece la generación de péptidos secundarios con efecto vasoconstrictor, proinflamatorio y de retención de sodio, que se relacionan con la fisiopatología de la hipertensión arterial.

La glicoproteína S del SARS-CoV-2 es el antígeno de superficie principal, con dos subunidades S1 y S2, de manera que la proteína distal S1 contiene el dominio de unión al receptor ACE2, lo que impide la actividad protectora natural de éste, mientras que la S2 contiene la subunidad de fusión de membrana para permitir la entrada del virus en la célula diana. Esta proteína S es la que provoca las respuestas inmunitarias durante la infección. La proteína de la nucleocápsida, que es el antígeno más conservado del virus, activa la señal AP1. Esta proteína es reconocida por las inmunoglobulinas en la fase aguda de la infección y por células T en la superficie de las células infectadas. La unión de la proteína S a ACE2 se activa por TMPRSS2, una proteína serina 2, así como por la catepsina [2], esta última probablemente facilitando la invaginación de la membrana y formación del endosoma requerido para la entrada el virus a la célula huésped [3]. El ARN viral, como PAMP, es detectado por los receptores PRRs, como TLR3, TLR7, TLR8 y TLR9. A partir de aquí, el gen I inducible por el receptor del ácido retinoico del virus (RIG-I), el gen 5 asociado a la diferenciación del melanoma (MDA5), y la nucleotidiltransferasa de la sintasa de GMP-AMP cíclico (cGAS), son responsables del reconocimiento del virus. La señalización intercelular incluye proteínas adaptadoras como la proteína adaptadora conteniendo el dominio TIR incluyendo INFβ (TRIF), proteína señalizadora antiviral mitocondrial (MAVS), y proteína estimulante de los genes de interferón (STING). Todas ellas disparan cascadas de moléculas que incluyen a la proteína adaptadora MyD88, que dan lugar a la activación del *factor nuclear potenciador de las cadenas ligeras kappa de las células B activadas* (NF-κB), que se desplaza al núcleo donde activa múltiples señales proinflamatorias y antioxidantes, dando lugar al aumento de las interleucinas (IL) IL-1, IL-2, IL-4, IL-6, IL-7, IL-10, IL-12, IL-13, IL-17, del *factor estimulante de colonias de granulocitos* (G-CSF), del

factor estimulante de colonias de macrófagos (M-CSF), de IP-10, de MCP-1, de MIP-1α, del *factor de crecimiento de hepatocitos* (HGF), del IFN-γ y del TNF-α, provocando una respuesta inmunológica exagerada que se asocia con la situación crítica de los pacientes de COVID-19 [4, 5].

El coronavirus infecta el tracto respiratorio, por gotículas o secreciones respiratorias y por contacto directo. No obstante, el hecho de que ACE2 se encuentra también en el epitelio intestinal y que el virus se haya aislado de las heces de algunos pacientes, indica que además de la vía respiratoria el virus puede infectar a través de la vía digestiva [6].

El método diagnóstico más eficaz es a través de una PCR a partir de muestras de exudado bronquial, nasofaríngea u orofaringea [7]; los métodos rápidos de diagnóstico se basan en la presencia de inmunoglobulinas IgM e IgG, que identifican que hubo un contagio, pero no que el virus esté activo en el organismo.

Los síntomas clínicos incluyen fiebre (88,7%), tos (67,8%), fatiga (38,1%), expectoración (33,4%), dificultad respiratoria (18,6%), y dolor de cabeza (13,6%). Además, parte de los pacientes muestran síntomas gastrointestinales, con diarrea (3,8%) y vómitos (5,0%), y otorrinolaringológicos como anosmia y ageusia (entre el 5% y el 19%, según los datos disponibles) [8, 9]. La susceptibilidad a la infección aumenta con la edad, hipertensión, enfermedad pulmonar obstructiva crónica, diabetes y enfermedad cardiovascular, mientras que las complicaciones más severas incluyen síndrome de distrés respiratorio, shock séptico, acidosis metabólica, alteración de la coagulación, y fallo multiorgánico [3, 6].

El análisis de laboratorio indica que la mayoría de los pacientes tiene el recuento de células blancas de la sangre normal o bajo, con linfocitopenia, aunque en los pacientes graves, el recuento de neutrófilos, la urea en sangre y los niveles de creatinina se elevan significativamente, mientras que los linfocitos continúan bajando. Además, los factores inflamatorios como IL-6, IL-10, y TNFα, se elevan, reflejando el estado de la inmunidad de los pacientes. Los pacientes en la UCI tienen mayores niveles de IL-2, IL-7, IL-10, GCSF, IP-10 (proteína inducida de 10 kD por IFN-γ), MCP-1, MIP-1α, y TNFα [6].

Las complicaciones incluyen síndrome de distrés respiratorio agudo (SDRA), arritmia, shock, fallo renal, fallo cardíaco, disfunción hepática, e infección secundaria y la enfermedad progresa más rápido en pacientes mayores, acortándose el número de días desde el primer síntoma hasta la muerte por encima de los 65 años [10-12].

Dado que no existe una terapia frente al SARS-CoV-2 efectiva, los tratamientos actuales son sintomáticos y de soporte respiratorio. Todos los pacientes aceptan la oxigenoterapia, y la Organización Mundial de la Salud (OMS) recomienda oxigenación extracorpórea (ECMO) en aquellos con hipoxemia refractaria [13]. Basados en la experiencia con otras epidemias virales (SARS-CoV y MERS-CoV), se han ensayado corticoides sistémicos y algunos antivirales, los inhibidores de la neuraminidasa (oseltamivir, peramivir, zanamivir, etc.), ganciclovir, aciclovir y ribavirina, pero sin resultados. Remdesivir ha resultado efectivo en un caso en Estados Unidos (EEUU). Por su parte, la cloroquina puede inhibir la replicación viral dependiente de pH, y suprime TNF-α [14] e IL-6 [15], además de interferir con la glicosilación de los receptores celulares de SASR-CoV. El remdesivir (Gilead) se está empleando actualmente en un ensayo clínico en España pacientes con COVID-19, y se ha propuesto el uso de remdesivir y hidroxicloroquina conjuntamente frente a la infección por SARS-CoV-2 [3].

Shiu y cols. [16] fueron los primeros en recomendar la melatonina frente a la infección por coronavirus. En ese momento el SARS-CoV-1 había infectado a 5050 personas con 316 fallecidos en 26 países. Argumentaban que, debido a sus propiedades como potente antioxidante [17-20] con actividad antiinflamatoria [21], sus efectos protectores frente a infecciones virales demostradas en animales [22-24] y los efectos positivos de la melatonina frente a condiciones clínicas con fisiopatología similar al SARS [25] merecía la pena usar la melatonina en conjunción con otras terapias para tratar el SARS con la intención de incrementar la eficiencia de los fármacos convencionales y reducir la tasa de mortalidad [26]. Defendían que la melatonina es barata y exhibe un muy alto margen de seguridad y podría ofrecer un relevante beneficio para mejorar el estado clínico y reducir la muerte en pacientes con SARS.

Una década más tarde, con la llegada de la epidemia de Ébola, tres grupos de investigación diferentes recomendaron el uso de la melatonina para tratar el Ébola utilizando argumentos similares a los esgrimidos por Shiu [26-29]. En ese momento se había mostrado que en humanos la melatonina reducía el estado protrombótico [30] y que junto al SB-73 era superior al aciclovir frente al herpes-1 [31].

Es relevante reseñar que la infección por SARS-CoV-2 tiene predilección por los grupos de pacientes que exhiben como factor común una producción reducida de melatonina, edad mayor 50 años y en especial mayores 60 años [32, 33] y varones [34] y en aquellas enfermedades en las que la melatonina está de alguna manera involucrada en la enfermedad como son las enfermedades endocrino-metabólicas [35] y cardiovasculares [36, 37]. Por el contrario, la infección por SARS-CoV-2 respeta a los pacientes de edad pediátrica [38] en los que los niveles de melatonina en plasma son más altos que en los adultos [32, 39]**.** Según el CDC-China de 44.762 casos, 1% eran < 10 años y 1% entre 10 y 19 años, y no hubo ningún fallecimiento en <10 años vs. 8% en 70-79 años y 14,8% > 80 años [40]. En Wuhan, de una cohorte de 171 casos confirmados en niños únicamente ingresaron 3 pacientes pediátricos (1,7%) en la UCIP [41].

Adicionalmente, el COVID-19 grave se caracteriza por un estado hiperinflamatorio, oxidación y respuesta inmune exagerada que conduce a una tormenta de citoquinas y a fallo multiorgánico, principalmente SDRA [42] pero también fallo cardiovascular, renal y cerebral [12] y como se mostrará a continuación la melatonina es un potente antioxidante, antiinflamatorio e inmunomodulador.

La melatonina tiene una plétora de acciones que la hacen extraordinariamente eficaz en reducir la agitación subcelular inducida por la destrucción oxidativa de elementos intracelulares clave, los cuales, cuando dañados, comprometen las funciones de las células resultando en su desintegración vía apoptosis o necrosis que desencadenan el estado hiperinflamatorio y el fallo multiorgánico [43].

Por tanto, la acción de la **melatonina** parece apuntar directamente a los **eventos inmuno-inflamatorios** asociados con la infección grave por SARS-CoV-2 que afectan de forma selectiva a grupos afectados por el proceso de envejecimiento y afecciones con reducida producción de melatonina y/o elevado estrés oxidativo y disfunción mitocondrial y además ha mostrado ser un tratamiento eficaz en las infecciones virales [44] y en la sepsis [45].

En consecuencia, la alta eficacia de la melatonina como agente para reducir la enfermedad asociada las infecciones virales radicaría en su rango de efectos como agente antioxidante, antiinflamatorio, inmunoestimulante e inmunomodulador de los procesos extracelulares e

intracelulares, los cuales una vez activados por el virus conducirían, en ausencia de la adecuada y ordenada modulación, a una respuesta hiperinflamatoria, apoptosis celular y fallo multiorgánico, como es el caso de la infección grave por SARS-CoV-2 [42, 46].

Además, recientemente se ha identificado que la melatonina regula indirectamente la expresión del *ACE2*, *el receptor clave involucrado en la infección del coronavirus*, por lo que la melatonina podría inhibir indirectamente un proceso clave de la infección del SARS-CoV-2 [1, 47].

En resumen, desde un punto de vista patogénico la melatonina inhibiría indirectamente el proceso clave de la infección del coronavirus y además modularía y neutralizaría los efectos celulares negativos desencadenados por la infección del SARS-CoV-2 evitando o minimizando la gravedad propia del COVID-19 en los adultos.

1.2 MEDICAMENTO EN INVESTIGACIÓN: MELATONINA

1.2.1 Datos disponibles de eficacia y seguridad

La melatonina (N-acetil-5 metoxitriptamina) es una indolamina sintetizada a partir del triptófano vía serotonina. Inicialmente descubierta como un producto de la glándula pineal en el cerebro, se produce de manera rítmica con un pico de secreción o acrofase entre 2-4 am. Su síntesis está regulada por el reloj biológico y se encarga de la sincronización de los ritmos circadianos como el sueño/vigilia, actividad/reposo, neurotransmisores cerebrales, hormonales, metabólicos, etc. Pero se sabe hoy en día que la melatonina se produce también en la mayoría sino todos los órganos y tejidos de nuestra economía, por la misma vía de síntesis. A diferencia de la pineal, la síntesis de la esta melatonina llamada extrapineal no sigue un ritmo circadiano y se produce en concentraciones mucho mayores que la de la pineal [48]. Estas elevadas concentraciones intracelulares de melatonina la dotan de un potencial antioxidante y antiinflamatorio muy eficaz, al tiempo que actúa como protector mitocondrial promoviendo la producción de ATP para la defensa celular. Su capacidad antioxidante es doble: por un lado, es un depurador directo de radicales libres, pero además induce la expresión y actividad de enzimas antioxidantes (superóxido dismutasa, catalasa, glutation peroxidasa y reductasa, y glucosa-6-fosfato deshidrogenasa), así como promueve la síntesis de glutation, uno de los principales antioxidantes intracelulares, reduciendo los marcadores de daño oxidativo como la peroxidación lipídica [49, 50].

La actividad antiinflamatoria de la melatonina depende de su doble capacidad para inhibir la respuesta de una respuesta exagerada de la inmunidad innata dependiente de NF-kB y del inflamosoma NLRP3, como la que se produce en procesos inflamatorios como la sepsis [51]. La melatonina también activa las células NK y disminuye el número total de polimorfonucleares circulantes [49]. Esta eficacia antiséptica de la melatonina ha llevado a utilizar un inyectable de melatonina patentado por nosotros en un ensayo clínico en pacientes sépticos (EudraCT nº 2008-00-6782-83) a los que se les administró por vía endovenosa, con unos notables resultados: reducción de la mortalidad al 0% (normalmente un 25%) y reducción de la estancia hospitalaria en un 43%.

Frente a las infecciones virales, la melatonina actúa como un potente agente antiviral en modelos experimentales. Al igual que en la sepsis, en infecciones virales severas como las producidas por SARS-CoV, SARS-CoV-2, MERS, Ébola y otros virus relacionados, la respuesta

inmunológica puede producir más daño que beneficio. En 2012, una revisión de Boga y cols. [44] resume los mecanismos antivirales de la melatonina en diferentes infecciones experimentales, debido a sus efectos para promover la vigilancia inmunitaria y eliminar radicales libres, reduciendo el daño celular. En el caso del virus del ébola, la infección promueve la lesión del endotelio vascular y la coagulación intravascular diseminada (CID). La lesión del endotelio se debe a una cascada de reacciones inflamatorias, activación de macrófagos, monocitos y liberación de citoquinas con generación excesiva de radicales libres de oxígeno y nitrógeno.

Las propiedades de la melatonina ya comentadas modulan las respuestas intracelulares exageradas y desordenadas que conducen a la apoptosis, estado hiperinflamatorio y CID [29]. En el caso del SARS-CoV-2, los mecanismos patogénicos involucrados incluyen su unión a ACE2 [1, 2]. Esta proteína protege al pulmón activando respuestas antiatróficas, antifibróticas, antioxidantes y antiinflamatorias. El virus deshabilita dichas funciones posibilitando su penetración en la célula y causando los síntomas respiratorios de COVID-19. Pero, además, al deshabilitar ACE2 se activa AT1R (receptor tipo 1 de angiotensina 2), el cual promueve respuestas proatróficas, profibróticas, prooxidantes y proinflamatorias, favoreciendo aún más el daño tisular. Una vez el SARS-CoV-2 ha frenado la defensa pulmonar, se activan las catepsinas, unas proteínas con actividad proteolítica, que favorecen la formación de endosomas y la entrada del virus en la célula [1].

Las propiedades antioxidantes y antiinflamatorias de la melatonina y su actividad para inhibir las catepsinas [52] sugieren un importante papel en la patogenia de la infección por SARS-CoV2 y en la evolución a COVID-19 grave. Por último, se ha realizado un estudio de su efectividad frente a las proteínas de envoltura y nucleocápside de 2019-nCoV/SARS-CoV-2. Usando análisis de proximidad de dianas farmacológicas e interacción HcoV-huésped en el interactoma humano, Zhou y cols. [47] han priorizado una serie de moléculas potenciales ante el virus SARS-CoV-2, principalmente la melatonina.

La melatonina tiene efectos protectores a todos los niveles. Específicamente a nivel pulmonar, la melatonina contrarresta el daño oxidativo y las complicaciones respiratorias de la edad, lo que mejora la función respiratoria, algo importante también en COVID-19 [53].

Un dato importante que ha surgido a lo largo de esta pandemia, es la relación entre infección por SARS-CoV-2, disfunción pulmonar y sepsis.

Por último, se ha demostrado la seguridad, eficacia y ausencia de efectos secundarios de la melatonina aún a distintas dosis y tras su administración intravenosa [54, 55].

1.2.2 Justificación de la selección de la dosis

Las propiedades de la melatonina para su administración en este ensayo clínico han sido sacadas de los distintos estudios en modelos experimentales, así como de datos clínicos anteriores. De esos estudios concluimos que los efectos antiinflamatorios y antioxidantes de la melatonina se ejercen a altas dosis de la misma. Estas dosis son debidas a que la melatonina, para ejercer su potencial antiinflamatorio y antioxidante, ha de entrar en la célula y ésta, según hemos demostrado, ejerce de una barrera que impide tanto la salida de la melatonina intracelular al exterior, como de la entrada de la melatonina exógena.

Las dosis se han obtenido de numerosos experimentos en modelos animales de sepsis y cáncer en nuestro Grupo de Investigación, que permitieron calcular la dosis equivalente humana,

que oscila entre 50 y 500 mg/día [56]. Aunque estas dosis son aproximadas, ya que en el ensayo clínico EudraCT: 2015-001534-13, la melatonina se aplicó en gel oral a dosis de 1500 mg/día, mientras que en el ensayo clínico EudraCT: 2008-006782-83, se usaron 60 mg de melatonina/día por vía intravenosa.

Con estas experiencias previas, y dada la gravedad de los pacientes de COVID-19, que muestran una reacción inflamatoria sistémica por la entrada del virus a la sangre, similar a la que ocurre en la sepsis, junto con la insuficiencia respiratoria que se produce a continuación, consideramos que la dosis de elección debe ser 5 mg/kg peso actual/día repartidos en 4 dosis al día (1 dosis/6h) y con una dosis máxima diaria de 500 mg.

1.2.3 Resumen de los riesgos y beneficios conocidos para los seres humanos

La utilidad terapéutica de la melatonina se ha demostrado en múltiples patologías, desde modelos experimentales a la clínica humana, con diferentes dosis y pautas de administración. En todos los casos los efectos han sido beneficiosos, con baja toxicidad [57]. Se ha usado para el tratamiento de la epilepsia infantil a dosis superiores a 100 mg/día [58]; en niños con distrofia muscular de Duchenne a dosis de 70 mg/día [59]; en pacientes con ELA a dosis de hasta 300 mg/día [60]; en sujetos controles con 100 mg/día [61], entre otros.

Ya han sido ensayadas en pacientes altas dosis de melatonina en dos ensayos clínicos de dos diferentes formulaciones: una, ensayo clínico en Fase II (**EudraCT: 2008-006782-83**) en pacientes de sepsis, a dosis de 60 mg iv/día durante 5 días, con resultados excelentes (reducción a cero la mortalidad y reducción de un 43% la estancia hospitalaria de los pacientes), y la segunda, otro ensayo clínico en Fase II (**EudraCT: 2015-001534-13**) con un gel oral de melatonina, a dosis de 1,5 gramos/día durante el tratamiento, frente a la mucositis en pacientes de cáncer de cabeza y cuello tratados con quimio y/o radioterapia, también con excelentes resultados. En ningún caso se observaron efectos secundarios a nivel hepático, renal, cardiovascular y metabólico. Numerosos estudios avalan la seguridad y ausencia de efectos secundarios de la melatonina a dosis altas.

Por tanto, los beneficios ya constatados en su administración a distintos tipos de pacientes a dosis elevadas, particularmente en lo que respecta al estudio con administración intravenosa, no se han acompañado de riesgos para la salud de los mismos.

1.3 JUSTIFICACIÓN DEL ESTUDIO

Nuestro Grupo de Investigación tiene una larga trayectoria en el uso de la melatonina para dilucidar sus efectos, mecanismo de acción, y uso clínico de la misma. Tanto en animales de experimentación como en la clínica humana, hemos comprobado como el uso terapéutico de melatonina, a dosis adecuadas, es capaz de modular la respuesta inmunitaria. El problema es que estas enfermedades virales están autolimitadas por una respuesta inmunitaria adaptativa que depende de la proliferación celular y, por tanto, requiere varias semanas para desarrollarse. En este período de ventana los pacientes son vulnerables y la mortalidad es alta por la activación de la inmunidad innata y el estrés oxidativo concomitante. El control de la respuesta inmune innata y la

reducción de la inflamación durante este período aumenta la tolerancia de los pacientes y disminuye la mortalidad en la infección mortal por virus.

La melatonina es una molécula que tiene la capacidad de romper este círculo vicioso. La melatonina es un poderoso depurador de radicales libres para reducir el daño oxidativo de los tejidos y también es un agente antiinflamatorio efectivo para deprimir las "tormentas de citoquinas" derivadas de la activación de la vía de NF-κB y del inflamosoma NLRP3. Como resultado, la melatonina puede aumentar la tolerancia del huésped a los patógenos y ahorrar un tiempo precioso para que los pacientes desarrollen una respuesta inmune adaptativa y finalmente se recuperen del ataque de los patógenos. Además, la melatonina también promueve la respuesta inmune adaptativa al aumentar la proliferación de linfocitos T y las células B para generar anticuerpos específicos [62].

La administración de melatonina previene del shock séptico y fallo multiorgánico [63], inhibiendo a la expresión de la iNOS y protegiendo la mitocondria del efecto nocivo del exceso de óxido nítrico [64]. El efecto de la melatonina fue incluso mayor en animales viejos que jóvenes sometidos a endotoxemia [65]. En cualquier caso, la melatonina previno el fallo multiorgánico en diferentes tejidos como hígado, pulmón, corazón, diafragma, etc. [66]. Cuando analizamos los mecanismos de acción de la melatonina para explicar esos llamativos efectos antiinflamatorios, encontramos que, por un lado, la melatonina actúa activando la SIRT1 a través del control de la expresión de los genes reloj [67, 68], uniéndose al receptor nuclear RORα; la SIRT1, una deacetilasa, deacetila a NF-κB en el núcleo, impidiendo su unión al ADN y terminando así su activación inflamatoria. Pero, la otra vía de la inmunidad innata, el inflamasoma NLRP3, continúa activo madurando la pro-IL-1β producida por la unión de NF-κB al ADN, a su forma activa, manteniendo la respuesta inflamatoria. El inflamasoma NLRP3 se activa por la presencia de PAMPs entre otros, y el daño mitocondrial, inducido por la producción de radicales libres (ROS) dependientes de la vía de NF-κB. Estos ROS dañan la mitocondria, abren el poro de transición, y liberan los propios ROS y ADN mitocondrial al citosol, activando el inflamasoma NLRP3. La melatonina actúa como un potente protector mitocondrial, reduce los ROS, cierra el poro de transición, bloqueando al inflamasoma, que ya no puede activar IL-1β [51, 69]. A nivel mitocondrial, la melatonina es mucho más potente que cualquier otro antioxidante, lo que favorece su protección y la producción de ATP, lo que permite a la célula disponer de más ATP para sus funciones de reparación y protección [70].

Numerosos estudios han informado los efectos beneficiosos de la melatonina en las infecciones virales mortales en diferentes modelos animales y su eficacia terapéutica en pacientes con shock séptico, como se ha comentado arriba [27, 29, 44]. Todas estas acciones, junto al efecto de la melatonina para contrarrestar la disfunción respiratoria debido al estrés oxidativo con la edad [53], así como para proteger el miocardio del daño durante el envejecimiento y la sepsis [71, 72], hacen de esta molécula una potencial opción terapéutica en COVID-19.

2. OBJETIVOS DEL ESTUDIO

2.1 OBJETIVO PRINCIPAL

El objetivo principal de este estudio es evaluar si el tratamiento con melatonina i.v. reduce la mortalidad en pacientes con COVID-19 ingresados en UCI.

2.2 OBJETIVOS SECUNDARIOS

Los objetivos secundarios del estudio incluyen:

- Evaluar si el tratamiento con melatonina i.v. reduce la duración del ingreso en la UCI.
- Evaluar si el tratamiento con melatonina i.v. reduce la duración de la ventilación mecánica (VM).
- Evaluar si el tratamiento con melatonina i.v. se asocia a un aumento de días libres de VM.
- Evaluar si el tratamiento con melatonina i.v. se asocia a una reducción del número de fallos de órganos vitales, según la escala SOFA.
- Evaluar si el tratamiento con melatonina i.v. se asocia a una reducción de frecuencia y gravedad de los procesos trombo-embólicos causados por COVID-19.
- Evaluar si el tratamiento con melatonina i.v. se asocia a una reducción de la respuesta inflamatoria sistémica, definida por los niveles de ferritina, dímero-D, proteína C reactiva (PCR) o procalcitonina (PCT) e IL-6.
- Evaluar si el tratamiento con melatonina i.v. se asocia a mejora de parámetros hematológicos.
- Evaluar si el tratamiento con melatonina i.v. se asocia a mejora de parámetros bioquímicos.
- Evaluar si el tratamiento con melatonina i.v. se asocia a mejora de parámetros gasométricos.
- Describir los acontecimientos y reacciones adversas acontecidos durante el estudio.

3. DISEÑO DEL ESTUDIO Y EVALUACIONES

3.1 TIPO DE ESTUDIO

Ensayo clínico de fase II aleatorizado, doble-ciego, controlado con placebo y unicéntrico que incluirá 18 pacientes.

3.2 DISEÑO Y TRATAMIENTO DEL ESTUDIO

3.2.1 Periodo de selección

Se contempla un periodo de 1 mes para la selección e inclusión de los pacientes.

3.2.2 Periodo de tratamiento

A los pacientes incluidos en el estudio se les administrará el tratamiento asignado hasta 7 días, salvo que tenga lugar alguno de los siguientes acontecimientos:

- Alta del paciente de la UCI.
- Fallecimiento del paciente.
- Aparición de toxicidad inaceptable.
- Interrupción del tratamiento justificada según criterio del investigador.

Dada la gravedad de los pacientes a incluir, si el paciente cumple con los criterios de selección recibirá la primera administración del tratamiento del estudio el mismo día de su inclusión.

Tras los 3 primeros días de tratamiento tres médicos intensivistas a cargo del paciente decidirán si prolongar el tratamiento hasta el día 6 del estudio (total de 7 días de tratamiento) en función de la evaluación clínica del paciente:

- Si observan mejoría o signos clínicos de recuperación o de no progresión, objetivos o subjetivos, del fallo respiratorio, del estado general del paciente, del estado inflamatorio o del fallo multiorgánico prolongarán el tratamiento hasta el día 6 del estudio (total de 7 días de tratamiento).
- Si observan algún acontecimiento adverso (AA) o empeoramiento achacable objetiva o subjetivamente al tratamiento del estudio lo suspenderán.

En cada caso, dejarán constancia de su valoración y decisión en la historia clínica del paciente.

3.2.3 Periodo de seguimiento

Una vez administrada la última dosis del tratamiento del estudio, a todos los pacientes se les hará una evaluación de seguridad a los 30 días.

3.2.4 Tratamiento del estudio

Tratamiento experimental: melatonina 6 mg/mL solución inyectable y para perfusión.

Control: placebo i.v.

Todos los pacientes incluidos recibirán el tratamiento *standard-of-care* (SOC) definido por el protocolo vigente en el centro en el momento del inicio del estudio y serán asignados al azar a uno de los siguientes grupos de tratamiento:

- **Grupo experimental**: SOC + melatonina por vía i.v. (5 mg/kg peso actual/día repartidos en 4 dosis al día [1 dosis/6h] y con una dosis máxima diaria de 500 mg).
- **Grupo control**: SOC + placebo por vía i.v.

3.2.5 Asignación al grupo de tratamiento

A cada paciente incluido en el estudio se le asignará un número de identificación de participante. Este número exclusivo se deberá utilizar en toda la documentación y correspondencia relacionada con este paciente en particular. A los pacientes elegibles, además del número de identificación, se les asignará un código de aleatorización enmascarado.

Personal de TFS ajeno al estudio, preparará los códigos de aleatorización desenmascarados, diseñados para que los pacientes sean asignados en proporción 2:1 al grupo experimental o al grupo control respectivamente. También preparará los sobres cerrados que contienen el código de aleatorización desenmascarado.

Al equipo investigador se les proporcionará un listado con códigos de aleatorización enmascarado, para que el personal investigador vaya asignando a cada paciente elegible, según orden de inclusión, un código de aleatorización enmascarado.

Durante el estudio los investigadores dispondrán de los sobres cerrados que contienen el código de aleatorización desenmascarado, para que el investigador pueda abrir el sobre y desenmascarar el tratamiento de un determinado paciente en caso de necesidad. En el apartado 10.2 se describen las circunstancias para abrir el ciego.

3.3 TERAPIAS CONCOMITANTES PROHIBIDAS O RESTRINGIDAS

3.3.1 Terapias prohibidas

No se contemplan medicaciones concomitantes prohibidas en este estudio, más allá de las incluidas en los criterios de selección.

3.3.2 Terapias restringidas y precauciones

No se han descrito.

3.4 VARIABLES DEL ESTUDIO

3.4.1 Variable principal

Mortalidad:

- Frecuencia absoluta y relativa.
- Tiempo (días) hasta el evento.

3.4.2 Variables secundarias

- Tiempo de ingreso en UCI antes del inicio del estudio (en días).
- Tiempo total de ingreso en UCI (en días).
- Tiempo total de ingreso hospitalario (en días).
- Tiempo con ventilación mecánica antes del inicio del estudio (en días).
- Tiempo libre de ventilación mecánica (en días).
- Cambio en la puntuación en la escala SOFA (*Sequential Organ Failure Assessment*) a los días 1, 3, 7, 14, 21 y 28.

- Numero de eventos, localización y gravedad (órgano y territorio vascular afectado) de eventos trombo-embólicos causados por COVID-19.
- Cambio en la respuesta inflamatoria sistémica evaluada a partir de la variación en los niveles de ferritina, dímero-D, proteína C reactiva (PCR) o procalcitonina (PCT) e IL-6 a los días 1, 3, 7, 14, 21 y 28.
- Cambio en los parámetros hematológicos evaluados a partir de la variación en Hemograma y Hb libre y en los niveles de plaquetas, fibrinógeno, Tº Cefalina, Tº Protrombina, Antitrombina III, ADAMTS13 (Ac Anti ADAMTS13 si ADAMTS < 20%), Factor Xa a los días 1, 3, 7, 14, 21 y 28.
- Cambio en los parámteros bioquímicos evaluados a partir de la variación en los niveles de CK, LDH, GOT, GPT, bilirrubina, Vit D y 1,25-OH-Vit D (calcitriol), calcio, albumina, BUN, creatinina y mioglobina a los días 1, 3, 7, 14, 21 y 28.
- Cambio en los parámetros gasométricos arteriales y electrolitos evaluados a partir de la variación en los valores de pH, SaO_2, $PaCO_2$, PO_2, HCO_3, glucosa, Na, K, Cloro, Ca iónico, lactato, anión gap, PaO_2/FiO_2 y puntuación en la escala MURRAY en los días 1, 3, 7, 14, 21 y 28.
- Incidencia de acontecimientos y reacciones adversas en pacientes adultos hospitalizados con COVID-19.

4. SELECCIÓN DE PACIENTES

4.1 POBLACIÓN DEL ESTUDIO

Los pacientes para el estudio se seleccionarán de entre aquellos con diagnóstico confirmado de COVID-19 que presenten criterios de ingreso en UCI, procedentes de Urgencias o de hospitalizados en planta, o bien de entre los pacientes ya ingresados en la UCI que cumplan estos criterios.

4.2 CRITERIOS DE INCLUSIÓN

Para participar en el estudio, los pacientes deberán cumplir TODOS los criterios de inclusión.

1. Otorgamiento de consentimiento informado por parte del paciente, su familiar o su representante legal.
2. Edad igual o superior a 18 años.
3. Presencia de infección por SARS-CoV-2, con clínica compatible y confirmación mediante PCR.
4. Ingreso en UCI con insuficiencia respiratoria aguda hipoxémica atribuidos a la infección por SARS-CoV-2
5. Llevar ingresado en UCI menos de 7 dias con o sin VM y sin signos de mejoría de la insuficiencia respiratoria (puntuación de MURRAY igual o superior a la puntuación presentada al ingreso en UCI).

4.3 CRITERIOS DE EXCLUSIÓN

Para participar en el estudio, los pacientes no deberán presentar NINGÚN criterio de exclusión.

1. Estar incluido en otro estudio de COVID-19 que implique la administración de un medicamento en fase de desarrollo clínico que carezca de una autorización de comercialización previa.
2. Transaminasas hepáticas > 5 veces el LSN.
3. Insuficiencia renal de estadio IV o en diálisis (GFR <30).
4. Embarazo. Se realizará un test de embarazo en toda mujer elegible menor 55 años antes de su inclusión.
5. Enfermedad terminal médica o quirúrgica.
6. Enfermedad autoinmune.
7. Cualquier condición del paciente que en opinión del investigador impida llevar a cabo los procedimientos del estudio.

5. DETALLE DE LOS PROCEDIMIENTOS DEL ESTUDIO

5.1 EVALUACIONES DE EFICACIA Y SEGURIDAD

Para realizar las evaluaciones de eficacia y seguridad en el contexto del estudio se llevarán a cabo los siguientes procedimientos:

- Consulta de la historia clínica.
- Medición de constantes vitales.
- Exploración física.
- Aplicación de escalas de valoración clínica.
- Obtención de muestras de sangre y otras (p. ej. exudados).
- Realización de pruebas de imagen:
    - **Radiografías torax** en cada visita, el dia de inclusión en el estudio y al finalizar.
    - **TAC torácico con angioTAC**: Si el paciente lo permite, idealmente el dia de inclusión en el estudio. En todo caso 1 TAC en la primera semana de la incorporación al estudio y un TAC en la visita final del estudio. Los TAC torácicos con angioTAC realizados en las 72 h previas a la inclusión del paciente o en las 48 h anteriores o posteriores a inicio de la VM o del ingreso en la UCI se considerarán válidos a efectos de TAC inicial.
- Realización de ecocardiograma.

Evaluación principal: mortalidad en cada grupo de tratamiento, en frecuencias y en tiempo hasta el evento.

Evaluaciones secundarias de eficacia: tiempo de ingreso en UCI, tiempo con ventilación mecánica y modificación del estado de gravedad del paciente respecto a los valores basales:

- Manifestaciones clínicas.

- Escala SOFA.
- Escala de Glasgow.
- Escala APACHE II.
- Escala ordinal de mejoría clínica de la OMS.
- Parámetros inflamatorios:
  - PCR, PCT, troponina, Dímero-D, IL-6, ferritina.
- Parámetros de coagulación:
  - Plaquetas, fibrinógeno, Tº Cefalina, Tº Protrombina, Antitrombina III, ADAMTS13 (Ac Anti ADAMTS13 si ADAMTS < 20%), Factor Xa.
- Parámetros bioquímicos:
  - CK, LDH, GOT, GPT, bilirrubina, Vit D y 1,25-OH-Vit D (calcitriol), calcio, albumina, BUN, creatinina, mioglobina.
- Parámetros hematológicos:
  - Hemograma, Hb libre
- Parámetros gasométricos arteriales y electrolitos:
  - pH, SaO_2, $PaCO_2$, PO_2, HCO_3, glucosa Na, K, Cloro, Ca iónico, Hb, lactato, anión gap, PaO_2/FiO_2.
- Escala MURRAY.

Evaluación de la seguridad:

- Acontecimientos adversos emergentes.
- Acontecimientos adversos emergentes relacionados con el tratamiento.
- Acontecimientos adversos emergentes por graduación de intensidad.
- Acontecimientos adversos emergentes graves (relacionados y no relacionados con el tratamiento).
- Acontecimientos adversos emergentes que llevan a la interrupción del estudio.
- Acontecimientos adversos emergentes con resultado de muerte.

Demografía y otras evaluaciones:

- Datos demográficos y antropométricos:
  - Edad (años)
  - Género
  - Peso (en kg)
  - Talla (en cm)
- Antecedentes personales:
  - Tabaquismo
  - Obesidad
  - Enfermedades previas (diabetes, hipertensión, enfermedad cardiovascular, etc)
- Manifestaciones clínicas y diagnóstico:
  - Fecha de inicio de los primeros síntomas de COVID-19
  - Manifestaciones clínicas
  - PCR:
    - Fecha del resultado positivo (DD/MM/AAAA) y origen de la muestra (nasofaríngeo, orofaringeo, aspirado bronquial, BAL).

        - Fecha de la negativización (DD/MM/AAA) y origen de la muestra (nasofaríngeo, orofaringeo, aspirado bronquial, BAL).
    - Test de detección de IgG e IgM:
        - Fecha seropositividad SARS-CoV-2 (DD/MM/AAAA).
    - Superinfección:
        - Germen/es.
        - Hemocultivos.
        - BAL.
        - Virus respiratorios.
- Registro de tratamientos concomitantes (dosis y fecha inicio):
    - Antivirales.
    - Corticoides sistémicos.
    - Anticoagulantes.
    - Insulina.
    - Hemoderivados: Concentrado de Hematíes, Plasma, Plaquetas, Antitrombina III.
    - Plasmaféresis.
    - Antihipertensivos.

5.2 PRUEBAS ANALÍTICAS

Las pruebas analíticas necesarias para el estudio se realizarán en el Laboratorio de Análisis Clínicos del centro participante.

Tabla 1. Pruebas analíticas

PRUEBA	CARACTERÍSTICAS
**Hemograma**	Serie blanca, serie roja, plaquetas
**Bioquímica**	Proteína C reactiva (PCR), procalcitonina (PCT), ferritina, troponina, CK, LDH, GOT, GPT, bilirrubina, Hb libre, calcio total, albumina, BUN, creatinina, mioglobina, IL-6*, dímero-D.
**Vit D***	Vit D y 1,25-OH-Vit D (calcitriol)
**Coagulación**	Hemograma, Fibrinógeno, Tº Cefalina, Tº Protrombina, Antitrombina III*, Factor Xa*
**ADAMTS13***	Si el paciente ha recibido transfusión plasma esperar al menos 72 h para realizar la extracción. Si el valor es menor 20% solicitar Ac anti ADAMTS13
**Gasometría arterial y electrolitos**	pH, SaO_2, $PaCO_2$, PO_2, HCO_3, Na, K, Cloro, Ca iónico, Hb, lactato, *anión gap*, PaO_2/FiO_2

***** parámetros que únicamente se pueden realizar entre semana en horario de mañana, se realizarán el día más próximo anterior o posterior a la visita correspondiente en que sea posible realizarlo.

5.3 ESQUEMA TEMPORAL DE LOS PROCEDIMIENTOS

Visita de selección + Tratamiento (Día 0)*

Se llevarán a cabo los siguientes procedimientos:

- Firma/otorgamiento de consentimiento informado
- Evaluación de los criterios de selección
- Test de embarazo, en caso de mujeres < 55 años

Si el paciente/familiar/representante legal otorga el consentimiento a participar y el paciente cumple los criterios de selección establecidos en este protocolo, el paciente será aleatorizado a un grupo de tratamiento y se continuará con los siguientes procedimientos:

- Anamnesis.
- Medicación concomitante.
- Registro de transfusiones de hemoderivados.
- Exploración física.
- Constantes vitales.
- Evaluación basal de APACHE; SOFA, VM, PaO_2/FiO_2 y escala MURRAY.
- Obtención de muestras de sangre para hemograma, bioquímica, gasometría, electrolito, coagulación, vitamina D y metabolitos derivados y ADAMTS 13.
- Realización de ecocardiograma y pruebas radiológicas (Rx y TAC torácico con angioTAC, este último a realizar dentro de la 1ª semana e idealmente en las primeras 48 h).

El paciente recibirá la primera dosis del tratamiento del estudio.

*El día de la primera administración de la medicación del estudio, se contabilizará como día 0 del estudio en el caso que este día no coincida con la selección.

Visita 1 (Día 1)

Se llevarán a cabo los siguientes procedimientos:

- Exploración física.
- Constantes vitales.
- Medicación concomitante.
- Registro de transfusiones de hemoderivados.
- Evaluación de AA.
- Evaluación de SOFA, VM, PaO2/FiO2 y escala MURRAY.
- Obtención de muestras de sangre para hemograma, bioquímica, gasometría, electrolito, coagulación, vitamina D y metabolitos derivados y ADAMTS 13.
- Realización de ecocardiograma y pruebas radiológicas (Rx y TAC torácico con angioTAC si no se ha realizado previamente).
- Administración del tratamiento del estudio correspondiente.

Visita 2 (Día 3)

Se llevarán a cabo los siguientes procedimientos:

- Exploración física.
- Constantes vitales.
- Medicación concomitante.
- Registro de transfusiones de hemoderivados.
- Evaluación de AA.
- Evaluación de SOFA, VM, PaO_2/FiO_2 y escala MURRAY.
- Obtención de muestras de sangre para hemograma, bioquímica, gasometría, electrolito, coagulación, vitamina D y metabolitos derivados y ADAMTS 13.
- Realización de pruebas radiológicas (Rx y TAC torácico con angioTAC si no se ha realizado previamente).
- Administración del tratamiento del estudio correspondiente.

En esta visita se realizará una valoración clínica por parte del equipo médico según los criterios especificados en la Sección 3.2.2 de este protocolo. Si se decide que el paciente debe continuar con el tratamiento de estudio, se le administrará el tratamiento hasta el Día 6 del estudio (total de 7 días de tratamiento).

Visita 3 (Día 7)

Se llevarán a cabo los siguientes procedimientos:

- Exploración física.
- Constantes vitales.
- Medicación concomitante.
- Registro de transfusiones de hemoderivados.
- Evaluación de AA.
- Evaluación de SOFA, VM, PaO_2/FiO_2 y escala MURRAY.
- Obtención de muestras de sangre para hemograma, bioquímica, gasometría, electrolito, coagulación, vitamina D y metabolitos derivados y ADAMTS 13.
- Realización de pruebas radiológicas (Rx y TAC torácico con angioTAC si no se ha realizado previamente).

Visita 4 (Día 14)

Se llevarán a cabo los siguientes procedimientos:

- Exploración física.
- Constantes vitales.
- Medicación concomitante.
- Registro de transfusiones de hemoderivados.
- Evaluación de AA.
- Evaluación de SOFA, VM, PaO_2/FiO_2, escala MURRAY y escala ordinal OMS.
- Obtención de muestras de sangre para hemograma, bioquímica, gasometría, electrolito, coagulación, vitamina D y metabolitos derivados y ADAMTS 13.
- Realización de pruebas radiológicas (Rx).

Visita 5 (Día 21)

Se llevarán a cabo los siguientes procedimientos:

- Exploración física.
- Constantes vitales.
- Medicación concomitante.
- Registro de transfusiones de hemoderivados.
- Evaluación de AA.
- Evaluación de SOFA, VM, PaO_2/FiO_2, escala MURRAY y escala ordinal OMS.
- Obtención de muestras de sangre para hemograma, bioquímica, gasometría, electrolito, coagulación, vitamina D y metabolitos derivados y ADAMTS 13.
- Realización de pruebas radiológicas (Rx).

Visita Final

La visita final del estudio tendrá lugar el día del alta de la UCI, el día del exitus, el día de discontinuación prematura del tratamiento o a los 28 días de la inclusión y se llevarán a cabo los siguientes procedimientos:

- Exploración física.
- Constantes vitales.
- Medicación concomitante.
- Registro de transfusiones de hemoderivados.
- Evaluación de AA.
- Evaluación de SOFA, VM, PaO_2/FiO_2, escala MURRAY y escala ordinal OMS.
- Obtención de muestras de sangre para hemograma, bioquímica, gasometría, electrolito, coagulación, vitamina D y metabolitos derivados y ADAMTS 13.
- Realización de ecocardiograma y pruebas radiológicas (Rx y TAC torácico con angioTAC).

Visita de Seguimiento

La visita de seguimiento tendrá lugar a los 30 días de haber recibido la última dosis del tratamiento del estudio y se llevará a cabo el siguiente procedimiento:

- Evaluación de AA

Tabla 2. Esquema temporal de los procedimientos

PROCEDIMIENTO	SELECCIÓN (basal pre tto) + TRATAMIENTO[A] (Día 0)	VISITA 1[B] (Día 1)	VISITA 2[B] (Día 3) + valoración continuación tratamiento	VISITA 3[B] (Día 7)	VISITA 4[B] (Día 14)	VISITA 5[B] (Día 21)	VISITA FINAL[B,] (Día 28[C])	VISITA DE SEGUIMIENTO (a los 30 días de finalizar el tratamiento)
	EVALUACIONES DE ELEGIBILIDAD							
**Consentimiento Informado**	☑							
**Criterios de inclusión y exclusión**	☑							
**Anamnesis**	☑							
**Test embarazo[D]**	☑							
	EVALUACIONES DE SEGURIDAD							
**Evaluación del riesgo CV y antecedentes**	☑							
**Exploración física**	☑	☑	☑	☑	☑	☑	☑	
**Constantes vitales**	☑	☑	☑	☑	☑	☑	☑	
**Evaluación de los AA**		☑	☑	☑	☑	☑	☑	☑
**Medicación previa y actual**	☑	☑	☑	☑	☑	☑	☑	
**Registrar Transfusión de hemoderivados**	☑							
**Valoración si tratamiento del estudio hasta día 6**			☑					
	EVALUACIONES DE EFICACIA							
**APACHE**	☑							
**VM**	☑	☑	☑	☑	☑	☑	☑	
**PaO_2/FiO_2**	☑	☑	☑	☑	☑	☑	☑	

**SOFA**	☑	☑	☑	☑	☑	☑	☑	
**MURRAY**	☑	☑	☑	☑	☑	☑	☑	
**Escala ordinal mejoría clínica OMS**					☑	☑	☑	
	ANALÍTICA							
**Hemograma**	☑	☑	☑	☑	☑	☑	☑	
**Bioquímica**	☑	☑	☑	☑	☑	☑	☑	
**Vit D y calcitriol**	☑	☑	☑	☑	☑	☑	☑	
**Gasometría y electrolitos**	☑	☑	☑	☑	☑	☑	☑	
**Coagulación**	☑	☑	☑	☑	☑	☑	☑	
**ADAMTS 13 (si no ha recibido Plasma en las 72 h antes)**	☑	☑	☑	☑	☑	☑	☑	
	OTRAS EVALUACIONES							
**Rx torax**	☑	☑	☑	☑	☑	☑	☑	
**ECOcardiografía**	☑	☑					☑	
**TAC torácico con angioTAC**	☑ (en la primera semana si el paciente lo permite. Idealmente primeras 48h)						☑	
	SUMINISTROS DEL FÁRMACO							
**Aleatorización[D] y administración de la medicación del estudio**	☑[E]	☑[F]	☑[G]					

A. Dada la gravedad de los pacientes a incluir, si el paciente cumple con los criterios de selección recibirá la primera administración del tratamiento del estudio ese mismo día.

B. En caso de permanecer en UCI, las visitas se realizarán en la UCI.

C. La visita final del estudio tendrá lugar el día del alta de la UCI, el día del exitus, el día de la discontinuación prematura del tratamiento o a los 28 días de la inclusión.

D. Únicamente en el Día 0.

E. El día de la primera administración de la medicación del estudio, se contabilizará como día 0 del estudio en el caso que este día no coincida con la selección.

F. La medicación se administrará también en el día 2 del estudio.

G. En función de la valoración clínica del paciente, la medicación se administrará también en los días 4, 5 y 6 del estudio.

5.4 FIN DEL ESTUDIO Y PERÍODO DE SEGUIMIENTO

5.4.1 Fin del estudio

El estudio termina con el último paciente completando el seguimiento de seguridad de 30 días después del fin del tratamiento. Se hará un esfuerzo razonable para hacer un seguimiento de los pacientes que han experimentado efectos adversos, hasta la resolución de estos eventos, pero no más de 30 días después del final de tratamiento del último paciente.

Al finalizar el estudio, se procederá a la apertura de ciego, para posterior análisis de datos que se recopilarán en el informe final de resultados del estudio.

5.4.2 Duración del seguimiento tras fin de estudio

Aquellos pacientes que permanezcan ingresados en la UCI u hospitalizados tras finalizar el estudio, es decir tras 30 días posteriores al final del tratamiento, serán seguidos para evaluar la duración total de su estancia en UCI, la duración de su ingreso hospitalario, tiempo hasta fallecimiento si ocurriera durante su ingreso en UCI u Hospital. El seguimiento se hará mediante revisión de historia clínica de los pacientes por parte del equipo investigador. Si los datos de seguimiento se obtienen con posterioridad a la presentación del informe final de resultados del estudio a las autoridades sanitarias, los datos de seguimiento se presentarían una vez disponibles como anexo al informe final de resultados presentado.

6. MEDICACIÓN DEL ESTUDIO

6.1 IDENTIFICACIÓN DE LA MEDICACIÓN DEL ESTUDIO

NOMBRE DE LA MEDICACIÓN	PRESENTACIÓN
Melatonina	Ampolla de 10 mL de solución inyectable y para perfusión intravenosa<br>Cada mL de solución contiene 6 mg de melatonina (total: 60 mg de melatonina por ampolla).
Placebo	Ampolla de 10 mL de la misma solución para perfusión intravenosa, sin melatonina.

La presentación externa de la medicación del estudio será indistinguible, de forma que las ampollas de melatonina y placebo tendrán un aspecto idéntico tanto de continente como de contenido para conservar el carácter doble ciego del estudio.

6.2 SUMINISTRO

El Promotor proporcionará la medicación del estudio que se utiliza en el transcurso de este ensayo, melatonina y placebo.

La información actualizada sobre melatonina se incluye en la versión actualizada del Manual del Investigador correspondiente.

6.3 ENVASADO Y ETIQUETADO

La **melatonina** se presenta en ampollas conteniendo cada una de ellas 60 mg de melatonina en 10 mL de solución intravenosa.

El **placebo** se presenta en ampollas de 10 mL de la misma solución que la empleada en las ampollas de melatonina, pero sin melatonina.

Las etiquetas de los fármacos contendrán, al menos, la siguiente información:

- Identificación del ensayo.
- Identificación del promotor y del Investigador principal.
- Número de lote.
- Instrucciones de dispensación.
- Condiciones de conservación.
- Fecha de caducidad.

6.4 MANEJO Y DISPENSACIÓN DE LA MEDICACIÓN DEL ESTUDIO

La medicación del estudio se deberá guardar en un área segura de acuerdo con las normativas locales. Es responsabilidad del investigador asegurar que el fármaco experimental se dispense exclusivamente a los participantes en el estudio. El fármaco experimental se dispensará sólo en los centros oficiales del estudio y por el personal autorizado de acuerdo con las normativas locales.

El investigador se deberá asegurar que la medicación se guarda según las condiciones ambientales (temperatura, luz y humedad) determinadas por el promotor y definidas en el manual del investigador.

La medicación del estudio se deberá conservar a una temperatura ambiente, no mayor de 30°C.

6.5 ADMINISTRACIÓN DE LA MEDICACIÓN DEL ESTUDIO

La medicación del estudio será administrada por vía i.v. por el personal sanitario de la UCI a cargo del paciente. La melatonina se administrará siguiendo la pauta establecida en función del peso y registrando el momento de la administración.

- grupo experimental (melatonina):
    - 5 mg/kg peso actual/día repartidos en 4 dosis al día (1 dosis/6h) y con una dosis máxima diaria de 500 mg.
- grupo control (placebo):
    - 5 mg/kg/día repartidos en 4 dosis al día (1 dosis/6h).

La(las) ampolla(s) del tratamiento del estudio se diluye(n) en un **total 100 mL de solución salina, suero glucosalino o glucosado 5% comercial** (**las ampollas requeridas en 100 mL de SSF o SG5% comercial**) y se administra(n) al paciente mediante perfusión de 30 minutos. Se realizará una perfusión cada 6 horas.

6.6 ERRORES DE MEDICACIÓN Y SOBREDOSIS

En este estudio pueden producirse errores de medicación procedentes de la administración del fármaco incorrecto, en un momento incorrecto o a una dosis incorrecta.

Independientemente de si el error de medicación está acompañado o no por un AA, según determine el investigador, el error de medicación y si procede cualquier AA asociado se deberán documentar en la historia clínica del paciente. Para más información sobre errores de medicación y sobredosis consultar la sección 7.10.

6.7 REGISTROS DE LA MEDICACIÓN DEL ESTUDIO EN LOS CENTROS INVESTIGADORES

Debe asegurarse un registro actualizado de las existencias de la medicación del estudio en cada centro, en el que **conste la cantidad y destino del fármaco experimental**. Los registros o inventarios deberán cumplir con las normativas y directrices vigentes y deberán incluir la siguiente información:

- Cantidad recibida y colocada en el almacén.
- Cantidad actualmente almacenada.
- Número de ID de la etiqueta o número de lote y fecha de validez o de vencimiento.

- Fecha de cada anotación en el inventario del fármaco experimental e iniciales de la persona responsable de dicha anotación.
- Cantidad dispensada y devuelta, incluyendo el código de identificación del paciente.
- Pérdidas ajenas al estudio (por ejemplo, extravíos, desechos y roturas).
- Cantidad devuelta al promotor.
- Cantidad destruida en el centro del estudio, si procede.

El promotor proporcionará formularios para facilitar el control del inventario si el personal del centro investigador no ha establecido un sistema que cumpla con estos requisitos.

6.8 DEVOLUCIÓN Y DESTRUCCIÓN DE LA MEDICACIÓN DEL ESTUDIO

6.8.1 Devolución de la medicación

El Monitor responsable del estudio deberá identificar y conciliar la medicación no utilizada.

Cuando el estudio haya terminado, el centro coordinará con el Promotor la devolución de la medicación no utilizada, debidamente identificada. Tras ello el Promotor será el encargado de su destrucción, de acuerdo con sus protocolos internos y la legislación aplicable.

7. SEGURIDAD Y NOTIFICACIÓN DE ACONTECIMIENTOS ADVERSOS

7.1 DEFINICIONES

Un **acontecimiento adverso** (AA) se define como *cualquier incidencia perjudicial para la salud en un paciente o sujeto de estudio tratado con un medicamento, aunque no tenga necesariamente relación causal con dicho tratamiento.*

Un AA puede ser, por tanto, cualquier signo desfavorable y no intencionado (incluyendo un hallazgo anormal de laboratorio), síntoma o enfermedad temporalmente asociada con el uso de un medicamento, esté o no relacionado con el medicamento en cuestión.

Una **reacción adversa** (RA) es *toda reacción nociva y no intencionada a un medicamento relacionada con cualquier dosis administrada.* A diferencia de un AA, en el caso de una reacción adversa existe una sospecha de relación causal entre el medicamento en investigación y dicha reacción adversa.

Un **acontecimiento adverso grave (AAG)** o una **reacción adversa grave (RAG)** será cualquier acontecimiento adverso o reacción adversa que, a cualquier dosis:

- provoque la muerte.
- amenace la vida del paciente.
- requiera la hospitalización del paciente o prolongue una hospitalización existente.
- provoque invalidez o incapacidad permanente o importante, o
- dé lugar a una anomalía o malformación congénita.

A efectos de su notificación, se tratarán también como graves aquellas sospechas de acontecimiento adverso o reacción adversa que se consideren importantes desde el punto de vista médico, aunque no cumplan los criterios anteriores, incluyendo los acontecimientos médicos importantes que requieran una intervención para evitar que se produzca una de las consecuencias anteriormente descritas. Asimismo, se notificarán como graves todas las sospechas de transmisión de un agente infeccioso a través de un medicamento.

Una **reacción adversa inesperada (RAI)** es cualquier reacción adversa cuya naturaleza, intensidad o consecuencias no se corresponde con la información de referencia para el medicamento (ej. el manual del investigador en el caso de un medicamento en investigación no autorizado para su comercialización, o la ficha técnica del producto en el caso de un medicamento autorizado).

Una **reacción adversa grave e inesperada (RAGI)** será cualquier reacción adversa que cumpla simultáneamente lo anteriormente descrito para las RAG y las RAI.

7.2 RELACIÓN DE CAUSALIDAD ENTRE EL ACONTECIMIENTO ADVERSO Y EL MEDICAMENTO DEL ESTUDIO

La relación causal entre un acontecimiento adverso y el medicamento del estudio se definirá del siguiente modo, considerándose relacionados con el tratamiento del estudio los acontecimientos con una causalidad:

- **Definitiva:** hay una evidencia clara que sugiere una relación causal, y se pueden descartar otros posibles factores contribuyentes. El acontecimiento clínico (o evento clínico), incluidas anomalías en las pruebas de laboratorio, que se produce con una secuencia temporal plausible respecto a la administración del fármaco, y que no puede ser explicado por la enfermedad concurrente o por otros fármacos o sustancias químicas. La respuesta a la retirada del fármaco debe ser clínicamente plausible. El acontecimiento debe ser definitivo farmacológica o fenomenológicamente, utilizándose si es necesario el procedimiento de reexposición que debe ser positivo.
- **Probable**: hay evidencia que sugiere relación causal, y la influencia de otros factores es poco probable. Un acontecimiento clínico, incluidas anomalías en las pruebas de laboratorio, que se produce con una secuencia temporal razonable a la administración del fármaco, que es poco probable que pueda atribuirse a la enfermedad intercurrente o a otros fármacos o sustancias químicas, y que después de ser retirado el fármaco sigue una secuencia clínica razonable. No se requiere reexposición para completar esta definición.
- **Posible**: hay alguna evidencia que sugiere relación causal (p. ej., secuencia temporal razonable a la administración del fármaco). No obstante, otros factores pueden haber contribuido al acontecimiento (p. ej., condición clínica del paciente, otros acontecimientos concomitantes).

Se considerarán no relacionados con la medicación del estudio, aquellos acontecimientos con una causalidad:

- **Improbable:** un acontecimiento clínico, incluidas anomalías en las pruebas de laboratorio, con una relación temporal respecto a la administración del fármaco que hace improbable la relación de causalidad (p. ej., el acontecimiento no ocurrió dentro de una secuencia temporal razonable a la administración del fármaco), y en el que otros fármacos, sustancias químicas o enfermedad intercurrente proporcionan explicaciones plausibles (p. ej., condición clínica del pacientes, otros tratamientos concomitantes).

- **No relacionado:** el AA es completamente independiente de la administración del tratamiento del estudio, y/o existen evidencias de que el acontecimiento está relacionado definitivamente con otra etiología. Debe haber una etiología alternativa definitiva documentada por el clínico.

7.3 EVALUACIÓN DE LA INTENSIDAD DE UN ACONTECIMIENTO ADVERSO

La intensidad de un acontecimiento adverso se registrará como una de las siguientes::

- **Leve**: el AA no interfiere de manera significativa en el nivel normal de funcionamiento del paciente aunque el AA puede causar molestias.
- **Moderado**: el AA interfiere de algún modo en el funcionamiento del paciente pero no es peligroso para su salud. Es molesto y/o una incomodidad.
- **Severo**: el AA produce una interferencia significativa en el funcionamiento o incapacitación y/o es peligroso para el paciente.

Un AA leve, moderado o severo puede ser o no ser grave. Estos términos se utilizan para describir la intensidad de un acontecimiento específico. **Las obligaciones de notificación de AA a las autoridades reguladoras están determinadas por la evaluación de la gravedad del AA, no de la intensidad**.

7.4 RECOPILACIÓN DE LA INFORMACIÓN SOBRE ACONTECIMIENTOS Y REACCIONES ADVERSAS

Durante el desarrollo del ensayo clínico, los acontecimientos adversos se podrán obtener a través de la comunicación espontánea del paciente, o a través de preguntas abiertas y una exploración física del paciente. Con el fin de evitar un sesgo en la notificación, no se preguntará a los pacientes si han experimentado uno o más acontecimientos adversos concretos.

Todos los AA identificados se deberán anotar y describir en la historia clínica del paciente. Si se conoce, se deberá anotar el diagnóstico de la enfermedad o la alteración subyacente en vez de los síntomas individuales. Se deberá recoger la siguiente información de todos los AA:

- fecha (y hora) de inicio (y resolución).
- intensidad del acontecimiento.
- relación causal con el medicamento en estudio en opinión del investigador.
- tratamiento requerido para el AA.
- criterio de gravedad del AA.
- información sobre su resolución o desenlace.

Después de obtener el consentimiento por escrito del paciente para participar en el estudio, se recopilarán todos los AA, incluidos aquellos que no se consideren asociados con los procedimientos del ensayo clínico. **La recopilación de información sobre los AA deberá empezar a partir de la obtención del consentimiento informado.**

Los acontecimientos adversos clasificados como “graves” se deberán anotar como GRAVES en la página de AA del CRD y ser reportados en el formulario de AAG proporcionado al efecto, y requieren una tramitación y notificación urgente al promotor.

7.5 PROCEDIMIENTO PARA LA COMUNICACIÓN DE AAG

Todos los AA graves, independientemente de su relación con el fármaco experimental, se deberán notificar al promotor en el formulario de AAG por fax o por correo electrónico dentro de las 24 h siguientes a la notificación del investigador.

Si inicialmente sólo se dispone de información limitada, será necesario redactar informes de seguimiento. La información mínima que debe incluirse en el informe inicial de AAG es:

- código de identificación del paciente del estudio
- descripción del AA y criterio para ser calificado como AAG.
- tratamientos que ha recibido el paciente.
- evaluación de la causalidad por parte del investigador.
- identificación de quien reporta el AAG (investigador o su delegado).

La información de seguimiento que esté disponible a medida que el AAG evolucione, se deberán recopilar posteriormente y enviar inmediatamente usando el mismo procedimiento que se usó con el informe inicial de AAG.

Los datos de contacto para la notificación de AAG se pueden consultar en la sección 7.11.

7.6 NOTIFICACIÓN EXPEDITIVA DE RAGI

TFS notificará a la Agencia Española de Medicamentos y Productos Sanitarios (AEMPS) y a los órganos competentes de la CCAA todas las sospechas de **RAGI asociadas a los medicamentos en investigación, en un plazo máximo de de 15 días naturales** a partir del momento en que el promotor haya tenido conocimiento de la sospecha de reacción adversa.

Cuando la sospecha de RAGI haya ocasionado la **muerte** del paciente, o puesto en peligro su vida, el promotor informará a la AEMPS **en el plazo máximo de 7 días naturales** a partir del momento en que el promotor tenga conocimiento del caso. Dicha información deberá ser completada, en lo posible, en los ocho días siguientes.

Cuando las sospechas de reacciones adversas graves e inesperadas ocurran en un ensayo clínico doble ciego, se deberá desvelar el código de tratamiento de ese paciente concreto por personal representante del promotor a efectos de notificación. Siempre que sea posible, se mantendrá el carácter ciego para el investigador.

7.7 SEGUIMIENTO DE LOS AA

- Se realizará un seguimiento de los AA hasta su resolución o estabilización hasta 30 días tras finalizar el tratamiento.

Si cambia la gravedad de un AA en progreso o se detecta que existe una relación con el fármaco del estudio, se deberá realizar una notificación nueva de AA.

7.8 ANOMALÍAS ANALÍTICAS

Todos los valores analíticos que se obtengan como parte de la evaluación de seguridad del estudio se deberán anotar en las páginas de resultados analíticos del CRD. Además se deberán anotar,

como mínimo, las siguientes anomalías analíticas en las páginas de AA graves o no graves del CRD, según corresponda:

- Cualquier resultado analítico que cumpla los criterios de un acontecimiento adverso grave.
- Cualquier anomalía analítica que requiera que el paciente suspenda temporal o permanentemente el tratamiento experimental.
- Cualquier anomalía analítica que requiera que el paciente reciba una terapia correctiva específica.

Se prefiere que, siempre que sea posible, el investigador que lleve a cabo la notificación utilice el término clínico MedDRA en vez del término analítico.

7.9 EMBARAZO

Antes de iniciar el tratamiento se realizará un test de embarazo a toda mujer menor de 55 años.

Los investigadores deben instruir a las pacientes mujeres, para que informen de inmediato si se quedan embarazadas hasta 30 días después de la última dosis administrada del fármaco en estudio.

Si, después de que una paciente haya iniciado el tratamiento del estudio se descubre que la mujer está embarazada o podría haber estado embarazada en el momento de la exposición al fármaco experimental, éste se suspenderá permanentemente de la forma adecuada. Se podrían realizar excepciones en cuanto a la suspensión del tratamiento del estudio, en caso de peligro vital para la paciente tras evaluación y decisión por parte del Comité Científico del estudio. **El investigador deberá anotar el embarazo en el Formulario del Embarazo y notificará al monitor y al promotor/representante del promotor este acontecimiento en el mismo plazo y siguiendo la misma ruta que para un AAG.**

7.10 SOBREDOSIS Y ERRORES DE MEDICACIÓN

Una sobredosis se define como la ingestión accidental o intencionada de cualquier dosis de un fármaco que se considere excesiva y clínicamente importante. Con propósitos de notificación, se considera una sobredosis, independientemente del desenlace adverso, como un acontecimiento médico importante (consulte la definición de acontecimientos adversos graves en la sección 7.1).

Se considera error de medicación el fallo por acción u omisión en el proceso de tratamiento con medicamentos que ocasiona o puede ocasionar un daño en el paciente. Los errores de medicación que ocasionen un daño en el paciente serán considerados a efectos de su notificación, como reacciones adversas, excepto aquellos derivados del fallo terapéutico por omisión del tratamiento.

7.11 RESPONSABLE DE LA NOTIFICACIÓN DE AAG

Los acontecimientos adversos graves (AAG) se recogerán y registrarán durante todo el período del estudio, comenzando a partir de la firma del consentimiento informado hasta 30 días después de la última dosis del producto en investigación o el final del estudio (incluido el período de seguimiento), lo que suceda más tarde.

La Unidad de Seguridad de Fármacos de TFS proporcionará los datos de contacto e instrucciones específicas sobre los AAG a todos los centros:

Detalles de contacto de la Unidad de Seguridad de Fármacos de TFS:

Fax: +46 (0) 46 280 19 19

e-mail: safety.tfs@tfscro.com

8. METODOLOGÍA ESTADÍSTICA

8.1 DETERMINACIÓN DEL TAMAÑO DE LA MUESTRA

Dada la naturaleza exploratoria del ensayo clínico, se realizará una aproximación muestral de estudio piloto, limitándose a 18 pacientes la muestra a incluir, con una proporción de asignación al grupo experimental o al grupo control de 2:1. Debido al pequeño tamaño de la muestra, los resultados obtenidos se interpretarán cautelosamente.

8.2 ANÁLISIS ESTADÍSTICO

Se utilizarán estadísticos descriptivos para resumir los datos analizados.

Los estadísticos descriptivos para las variables continuas numéricas incluirán número de valores observados, número de pacientes con datos ausentes (*missing*), media, desviación estándar (DE), intervalo de confianza del 95% (IC95%) de la media, mediana, primer cuartil (Q1), tercer cuartil (Q3) y valores mínimo y máximo. Para las variables categóricas, se presentará el número y porcentaje de pacientes (junto con IC95% del porcentaje para los criterios de valoración de eficacia binarios) con un valor específico de la variable o con *missing*.

Los datos de mortalidad se presentarán en frecuencias absolutas y relativas, y en días transcurridos hasta el evento. El análisis de eficacia se realizará para probar la superioridad del tratamiento experimental frente al placebo, mediante curvas de supervivencia (método de Kaplan-Meyer) y un modelo de riesgos proporcionales de Cox para comprobar si hubiera algún factor de riesgo que pudiera afectar la mortalidad.

Los resultados de laboratorio se resumirán por visita y por grupo de tratamiento.

Los cambios en los valores analíticos y las constantes vitales se resumirán con estadísticas descriptivas.

El análisis principal de seguridad resumirá todos los acontecimientos adversos que tengan lugar durante todo el ensayo. Las tasas de incidencia de acontecimientos adversos en los pacientes, los AEs relacionados con el tratamiento y los acontecimientos adversos graves y los acontecimientos adversos con resultado de muerte se presentarán en tablas descriptivas por tratamiento recibido.

La exposición al tratamiento se resumirá utilizando estadísticas de tipo descriptivo.

La medicación y tratamientos concomitantes recogidos en el estudio se tabularán de acuerdo con el código *Anatomical Therapeutic Chemical* (ATC) de nivel 3 y según término preferente del *WHO Drug Dictionary*.

Los datos de los pacientes individuales serán mostrados en listados.

Las poblaciones utilizadas para el análisis se definen a continuación:

- **Población de seguridad**: Todos los pacientes a los que se les haya administrado por lo menos una dosis de la medicación del estudio.
- **Población por intención de tratar:** Todos los pacientes que cumplen los criterios de selección y con al menos una medición de eficacia disponible después de la visita basal.

Dadas las características exploratorias del estudio, las variables principal y secundarias de eficacia se analizarán en la población por intención de tratar (ITT).

Las variables secundarias de seguridad se analizarán en la población de seguridad.

Las variables secundarias demográficas/clínicas se analizarán en la población ITT.

Una vez finalizado el protocolo, se escribirá un Plan de Análisis Estadístico (PAE), que proporcionará todos los detalles técnicos del análisis estadístico.

9. ASPECTOS ÉTICOS Y NORMATIVOS

9.1 CUMPLIMIENTO NORMATIVO

El estudio se realizará y registrará de acuerdo con el protocolo, las directrices de la Conferencia Internacional de Armonización (ICH) y los principios éticos estipulados en la Declaración de Helsinki en su última versión. El estudio también cumplirá cualquier normativa local vigente, incluida la Regulación (EU) 536/2014 del Parlamento Europeo y del Consejo sobre ensayos clínicos.

9.2 RESPONSABILIDAD CIVIL

Existe una póliza de responsabilidad civil que cubre cualquier daño que los pacientes puedan sufrir como resultado de su participación en este ensayo, de acuerdo con la normativa vigente en España (RD1090/2015 de 4 de diciembre), que ampara la responsabilidad civil legal del tomador del seguro, en su calidad de Promotor del ensayo, del investigador y de sus colaboradores, del hospital o centro donde se realice el ensayo y de sus titulares y que proporcionará la compensación e indemnización en caso de menoscabo de la salud de la paciente o de las lesiones que pudieran producirse en relación con la participación en una paciente en este ensayo.

Los pacientes serán informados de la existencia del seguro y las obligaciones resultantes de su parte.

9.3 CONSENTIMIENTO INFORMADO

Todo paciente incluido en el estudio deberá haber prestado previamente su consentimiento firmado. Para ello, los investigadores se deben asegurar que los pacientes, sus familiares o sus representantes legales estén clara y completamente informados sobre el objetivo, efectos esperables, posibles riesgos y cualquier otro aspecto relevante relacionado con el ensayos clínico antes de su participación. Se deberá informar a los pacientes del estudio de:

- que son libres de retirarse del mismo en cualquier momento y sin tener que dar explicaciones.

- el modo en que serán recogidos y utilizados los datos personales y los datos relacionados con la salud del paciente durante el estudio.

El proceso del consentimiento informado quedará documentado en la historia clínica o los registros médicos de cada paciente.

Todos los pacientes, o su familiar o su representante legal, recibirán una copia de la Hoja de Información al Paciente y una copia de su formulario de Consentimiento Informado firmado y fechado.

En caso de ser necesario, por imposibilidad para obtener el consentimiento del paciente debido a su estado y dada la situación vigente en España respecto a medidas de seguridad frente al coronavirus, se podrá recabar el consentimiento de un familiar o del representante legal verbalmente, mediante llamada telefónica grabada.

Todos los formularios de consentimiento firmados y fechados deben conservarse en el archivo del estudio de cada paciente y deben estar disponibles para su verificación por parte de los monitores del estudio en cualquier momento.

Se deberá revisar el formulario de consentimiento informado cuando se produzcan cambios en los procedimientos descritos en el consentimiento informado o cuando se obtenga nueva información que pueda afectar a la voluntad de la paciente para participar en el estudio.

En caso de que se actualice o revise el formulario de consentimiento en la historia clínica de cada paciente se documentará el proceso del consentimiento informado y se indicará que se ha obtenido el consentimiento informado por escrito actualizado/revisado para continuar participando en el estudio.

9.4 PROTECCIÓN DE DATOS

La información médica de pacientes obtenida en virtud de este estudio es confidencial y se encuentra amparada por el Reglamento (UE) nº 2016/679 General de Protección de Datos.

La confidencialidad del paciente se asegurará mediante la identificación únicamente por un código numérico asignado al inicio del estudio y nunca por nombre, iniciales, nº de historia clínica o cualquier otro dato personal identificable.

Durante la realización del estudio, se cumplirá asimismo con lo dispuesto por la Ley 41/2002, de 14 de noviembre, básica reguladora de la autonomía del paciente y de derechos y obligaciones en materia de información y documentación clínica, en lo que resulte de aplicación.

Los datos generados en este estudio deben estar disponibles para su inspección bajo petición de los representantes de las autoridades sanitarias nacionales y locales, los monitores del promotor, los representantes, los colaboradores y el CEIC de cada centro del estudio, según proceda.

En cualquier momento, el paciente podrá revocar el consentimiento informado para el tratamiento de sus datos personales, dirigiéndose directamente al investigador.

10. GESTIÓN DEL ESTUDIO

10.1 RETIRADA DE LOS PACIENTES DEL ESTUDIO

Los pacientes DEBERÁN SUSPENDER la terapia del estudio Y/O RETIRARSE del mismo por los siguientes motivos:

- Retirada del consentimiento informado (decisión del paciente de retirarse independientemente del motivo).
- Toxicidad inaceptable.
- Progresión de la enfermedad que, en opinión del investigador, no permita la continuidad del paciente en el estudio.
- El paciente no cumple con los requisitos del protocolo, con el tratamiento o con la monitorización.
- Cualquier otro motivo para interrumpir el tratamiento que, en opinión del investigador, sea lo mejor para el paciente.
- Cualquier acontecimiento adverso clínico, anomalía analítica o enfermedad intercurrente que, en la opinión del investigador, indique que continuar el tratamiento con dicha terapia y con la participación en el estudio no es lo mejor para el paciente.
- Terminación del estudio por parte del equipo investigador.

10.2 DESENMASCARAMIENTO Y APERTURA DEL CÓDIGO

La medicación del estudio se suministrará con idéntico aspecto de continente y contenido para preservar la naturaleza doble ciego del estudio.

No obstante, durante el estudio, se podrá abrir el código de aleatorización para desenmascarar el tratamiento de un determinado paciente si concurre alguna de las siguientes circunstancias:

- aparición de una urgencia médica siempre que para su tratamiento y toma de decisiones terapéuticas fuera necesario conocer la identidad del medicamento del estudio administrado.
- aparición de un AAG relacionado con el tratamiento o una RAGI que requieran de notificación expeditiva conforme a la normativa vigente.
- aparición de un AA que pueda requerir la retirada del paciente según el criterio del investigador, si dicha retirada estuviera condicionada por la relación causal con la medicación del estudio frente a la relación causal con los procedimientos o la enfermedad a estudio.

Toda apertura de código será registrada y se guardará una copia en el archivo del estudio. Deberá anotarse la fecha, la hora y el motivo de la apertura del código, debidamente firmado y fechado. La información pertinente sobre el desenmascaramiento del tratamiento de un paciente debe anotarse en la historia clínica del paciente.

En todos los casos el acceso y manejo de la información sin enmascaramiento estará limitada al menor número de personas posible.

10.3 MODIFICACIONES DEL PROTOCOLO

El estudio se llevará a cabo de la forma descrita en el protocolo aprobado por CEIm y AEMPS. El investigador no podrá desviarse o cambiar el protocolo sin la revisión previa y la aprobación/dictamen

favorable del CEIm de la correspondiente enmienda, excepto cuando sea necesario eliminar un peligro inmediato para los pacientes del estudio. Cualquier desviación significativa se deberá documentar en la historia clínica del paciente.

Si se realiza una desviación o cambio en un protocolo para eliminar un riesgo inmediato antes de obtener la aprobación/dictamen favorable del CEIm, tan pronto como sea posible dicha desviación o cambio se enviará a:

- CEIm para su revisión y aprobación/dictamen favorable.
- Autoridades reguladoras, si así lo indican las leyes locales.
- Al promotor.

11. GESTION Y CALIDAD LOS DE DATOS

11.1 MONITORIZACIÓN

La monitorizacíón del estudio será realizada por TFS.

Se deberá permitir al monitor visitar todas las instalaciones del centro involucradas en el estudio para evaluar los datos así como la calidad e integridad del mismo. En el centro, revisarán los archivos del estudio y los compararán directamente con los documentos fuente. También comentará el desarrollo del estudio con el investigador y se verificará que las instalaciones siguen siendo aceptables.

11.2 ARCHIVOS E INFORMES

El Investigador deberá preparar y mantener los historiales adecuados y exactos, diseñados para registrar todas las observaciones y otros datos pertinentes para la investigación de cada paciente tratado con el fármaco experimental o incluido como control en el ensayo. Los datos registrados en el cuaderno de recogida de datos (CRD), derivados de los documentos originales, deberán ser coherentes con dichos documentos originales, o en caso de discrepancias, éstas se deberán explicar.

El CRD electrónico, se completará de acuerdo con sus instrucciones específicas, <u>no debiéndose dar opción a dejar campos en blanco.</u>

Se deberá proteger la confidencialidad de aquellos documentos que pudieran identificar a los pacientes, respetando su intimidad y las reglas de confidencialidad de acuerdo con los requisitos reguladores aplicables.

11.3 RETENCIÓN DE ARCHIVOS

El investigador deberá conservar los inventarios de disponibilidad del fármaco del estudio, las copias de los CRDs (o archivos electrónicos), y los documentos originales durante el periodo máximo de tiempo que permitan las leyes y directrices vigentes, o los procedimientos de la institución o durante el periodo especificado por el promotor, el que sea más prolongado. El investigador se deberá poner en contacto con el promotor antes de destruir ningún archivo asociado con el estudio.

Si el investigador se retirase del estudio (p. ej., traslado, jubilación), los archivos se deberán transferir a otra persona cuya designación haya sido mutuamente acordada (por ejemplo, otro investigador, CEIC). La notificación de dicha transferencia se deberá enviar por escrito al promotor.

11.4 INSPECCIONES Y AUDITORÍAS

El promotor, ya sea directamente o a través de terceros contratados, podrá llevar a cabo auditorías de garantía de calidad en el centro. En caso de auditorías, el auditor tendrá acceso a las historias clínicas, a los archivos y a la documentación relevante relacionada con el ensayo clínico.

Las Autoridades Sanitarias pueden realizar inspecciones durante el estudio y tras su finalización. En caso de auditorías y/o inspecciones, el investigador facilitará toda la documentación relacionada con el estudio.

12. DIFUSIÓN DE RESULTADOS

La publicación de los resultados de este por parte de los investigadores o de un tercero se podrá realizar después de que se haya obtenido el consentimiento por escrito por parte del promotor. Todo material destinado a la publicación se entregará al promotor con antelación suficiente para su revisión antes de su presentación para su publicación.

13. INFORME FINAL DEL ESTUDIO

Una vez completada la visita final de estudio de todos los pacientes, la base de datos será bloqueada y todos los resultados que se hayan recopilado se tabularán, evaluarán y publicarán en forma de informe final del estudio clínico completo de acuerdo con la nota ICH-E3 para la orientación en la estructura y el contenido de informes de estudios clínicos. Los datos recopilados después del bloqueo de la base de datos se comunicarán en un informe de estudio actualizado.

El promotor enviará un resumen del informe final del estudio clínico al CEIm y a las autoridades competentes en el plazo de un año tras la finalización del ensayo.

14. BIBLIOGRAFÍA

1. Zhang H, Penninger JM, Li Y, Zhong N, Slutsky AS: **Angiotensin-converting enzyme 2 (ACE2) as a SARS-CoV-2 receptor: molecular mechanisms and potential therapeutic target**. *Intensive Care Med* 2020, **46**(4):586-590.
2. Hoffmann M, Kleine-Weber H, Schroeder S, Kruger N, Herrler T, Erichsen S, Schiergens TS, Herrler G, Wu NH, Nitsche A *et al*: **SARS-CoV-2 Cell Entry Depends on ACE2 and TMPRSS2 and Is Blocked by a Clinically Proven Protease Inhibitor**. *Cell* 2020.
3. Guo YR, Cao QD, Hong ZS, Tan YY, Chen SD, Jin HJ, Tan KS, Wang DY, Yan Y: **The origin, transmission and clinical therapies on coronavirus disease 2019 (COVID-19) outbreak - an update on the status**. *Military Medical Research* 2020, **7**(1):11.
4. Yingxia Liu CZ, Fengming Huang,Yang Yang,Fuxiang Wang,Jing Yuan,Zheng Zhang,Yuhao Qin,Xiaoyun Li,Dandan Zhao,Shunwang Li,Shuguang Tan,Zhaoqin Wang,Jinxiu Li,Chenguang Shen,Jianming Li,Ling Peng,Weibo Wu,Mengli Cao,Li Xing,Zhixiang Xu,Li Chen,Congzhao Zhou,William J. Liu,Lei Liu, Chengyu Jiang: **2019-novel coronavirus (2019-nCoV) infections trigger an exaggerated cytokine response aggravating lung injury**. *[ChinaXiv:20200200018]* 2020.
5. Liu Y, Yang Y, Zhang C, Huang F, Wang F, Yuan J, Wang Z, Li J, Li J, Feng C *et al*: **Clinical and biochemical indexes from 2019-nCoV infected patients linked to viral loads and lung injury**. *Science China Life sciences* 2020, **63**(3):364-374.
6. Huang C, Wang Y, Li X, Ren L, Zhao J, Hu Y, Zhang L, Fan G, Xu J, Gu X *et al*: **Clinical features of patients infected with 2019 novel coronavirus in Wuhan, China**. *Lancet (London, England)* 2020, **395**(10223):497-506.
7. Tang Y-W, Schmitz JE, Persing DH, Stratton CW: **The Laboratory Diagnosis of COVID-19 Infection: Current Issues and Challenges**. *Journal of Clinical Microbiology* 2020:JCM.00512-00520.
8. Mao L, Wang M, Chen S, He Q, Chang J, Hong C, Zhou Y, Wang D, Li Y, Jin H *et al*: **Neurological Manifestations of Hospitalized Patients with COVID-19 in Wuhan, China: a retrospective case series study**. *medRxiv* 2020:2020.2002.2022.20026500.
9. Vaira LA, Salzano G, Deiana G, De Riu G: **Anosmia and ageusia: common findings in COVID-19 patients**. *The Laryngoscope* 2020.
10. Zhou F, Yu T, Du R, Fan G, Liu Y, Liu Z, Xiang J, Wang Y, Song B, Gu X *et al*: **Clinical course and risk factors for mortality of adult inpatients with COVID-19 in Wuhan, China: a retrospective cohort study**. *Lancet (London, England)* 2020.
11. Zhang J-j, Dong X, Cao Y-y, Yuan Y-d, Yang Y-b, Yan Y-q, Akdis CA, Gao Y-d: **Clinical characteristics of 140 patients infected with SARS-CoV-2 in Wuhan, China**. *Allergy*, **n/a**(n/a).
12. Ruan Q, Yang K, Wang W, Jiang L, Song J: **Clinical predictors of mortality due to COVID-19 based on an analysis of data of 150 patients from Wuhan, China**. *Intensive Care Medicine* 2020.
13. World Health O: **Clinical management of severe acute respiratory infection when novel coronavirus (2019-nCoV) infection is suspected: interim guidance, 28 January 2020**. In. Geneva: World Health Organization; 2020.

14. Li R, Lin H, Ye Y, Xiao Y, Xu S, Wang J, Wang C, Zou Y, Shi M, Liang L *et al*: **Attenuation of antimalarial agent hydroxychloroquine on TNF-alpha-induced endothelial inflammation**. *International immunopharmacology* 2018, **63**:261-269.
15. Dewitte A, Villeneuve J, Lepreux S, Bouchecareilh M, Gauthereau X, Rigothier C, Combe C, Ouattara A, Ripoche J: **CD154 Induces Interleukin-6 Secretion by Kidney Tubular Epithelial Cells under Hypoxic Conditions: Inhibition by Chloroquine**. *Mediators of inflammation* 2020, **2020**:6357046.
16. Shiu SY, Reiter RJ, Tan DX, Pang SF: **Urgent search for safe and effective treatments of severe acute respiratory syndrome: is melatonin a promising candidate drug?** *Journal of pineal research* 2003, **35**(1):69-70.
17. Mohan N, Sadeghi K, Reiter RJ, Meltz ML: **The neurohormone melatonin inhibits cytokine, mitogen and ionizing radiation induced NF-kappa B**. *Biochemistry and molecular biology international* 1995, **37**(6):1063-1070.
18. Poeggeler B, Reiter RJ, Tan DX, Chen LD, Manchester LC: **Melatonin, hydroxyl radical-mediated oxidative damage, and aging: a hypothesis**. *Journal of pineal research* 1993, **14**(4):151-168.
19. Reiter RJ: **Oxygen radical detoxification processes during aging: the functional importance of melatonin**. *Aging (Milan, Italy)* 1995, **7**(5):340-351.
20. Tan DX, Manchester LC, Reiter RJ, Qi WB, Karbownik M, Calvo JR: **Significance of melatonin in antioxidative defense system: reactions and products**. *Biological signals and receptors* 2000, **9**(3-4):137-159.
21. Cuzzocrea S, Reiter RJ: **Pharmacological actions of melatonin in acute and chronic inflammation**. *Current topics in medicinal chemistry* 2002, **2**(2):153-165.
22. Bonilla E, Rodon C, Valero N, Pons H, Chacin-Bonilla L, Garcia Tamayo J, Rodriguez Z, Medina-Leendertz S, Anez F: **Melatonin prolongs survival of immunodepressed mice infected with the Venezuelan equine encephalomyelitis virus**. *Transactions of the Royal Society of Tropical Medicine and Hygiene* 2001, **95**(2):207-210.
23. Bonilla E, Valero N, Chacin-Bonilla L, Medina-Leendertz S: **Melatonin and viral infections**. *Journal of pineal research* 2004, **36**(2):73-79.
24. Bonilla E, Valero-Fuenmayor N, Pons H, Chacin-Bonilla L: **Melatonin protects mice infected with Venezuelan equine encephalomyelitis virus**. *Cellular and molecular life sciences : CMLS* 1997, **53**(5):430-434.
25. Gitto E, Karbownik M, Reiter RJ, Tan DX, Cuzzocrea S, Chiurazzi P, Cordaro S, Corona G, Trimarchi G, Barberi I: **Effects of melatonin treatment in septic newborns**. *Pediatric research* 2001, **50**(6):756-760.
26. Reiter RJ, Tan DX, Sainz RM, Mayo JC, Lopez-Burillo S: **Melatonin: reducing the toxicity and increasing the efficacy of drugs**. *The Journal of pharmacy and pharmacology* 2002, **54**(10):1299-1321.
27. Anderson G, Maes M, Markus RP, Rodriguez M: **Ebola virus: melatonin as a readily available treatment option**. *Journal of medical virology* 2015, **87**(4):537-543.
28. Masters A, Pandi-Perumal SR, Seixas A, Girardin JL, McFarlane SI: **Melatonin, the Hormone of Darkness: From Sleep Promotion to Ebola Treatment**. *Brain disorders & therapy* 2014, **4**(1).
29. Tan DX, Korkmaz A, Reiter RJ, Manchester LC: **Ebola virus disease: potential use of melatonin as a treatment**. *Journal of pineal research* 2014, **57**(4):381-384.

30. Wirtz PH, Spillmann M, Bärtschi C, Ehlert U, von Känel R: **Oral melatonin reduces blood coagulation activity: a placebo-controlled study in healthy young men**. *Journal of pineal research* 2008, **44**(2):127-133.
31. Nunes Oda S, Pereira Rde S: **Regression of herpes viral infection symptoms using melatonin and SB-73: comparison with Acyclovir**. *Journal of pineal research* 2008, **44**(4):373-378.
32. Grivas TB, Savvidou OD: **Melatonin the "light of night" in human biology and adolescent idiopathic scoliosis**. *Scoliosis* 2007, **2**:6.
33. Scholtens RM, van Munster BC, van Kempen MF, de Rooij SE: **Physiological melatonin levels in healthy older people: A systematic review**. *Journal of psychosomatic research* 2016, **86**:20-27.
34. Gunn PJ, Middleton B, Davies SK, Revell VL, Skene DJ: **Sex differences in the circadian profiles of melatonin and cortisol in plasma and urine matrices under constant routine conditions**. *Chronobiology international* 2016, **33**(1):39-50.
35. Cipolla-Neto J, Amaral FG, Afeche SC, Tan DX, Reiter RJ: **Melatonin, energy metabolism, and obesity: a review**. *Journal of pineal research* 2014, **56**(4):371-381.
36. Pechanova O, Paulis L, Simko F: **Peripheral and central effects of melatonin on blood pressure regulation**. *International journal of molecular sciences* 2014, **15**(10):17920-17937.
37. Sun H, Gusdon AM, Qu S: **Effects of melatonin on cardiovascular diseases: progress in the past year**. *Current opinion in lipidology* 2016, **27**(4):408-413.
38. Lee PI, Hu YL, Chen PY, Huang YC, Hsueh PR: **Are children less susceptible to COVID-19?** *Journal of microbiology, immunology, and infection = Wei mian yu gan ran za zhi* 2020.
39. Bubenik GA, Konturek SJ: **Melatonin and aging: prospects for human treatment**. *Journal of physiology and pharmacology : an official journal of the Polish Physiological Society* 2011, **62**(1):13-19.
40. Wu Z, McGoogan JM: **Characteristics of and Important Lessons From the Coronavirus Disease 2019 (COVID-19) Outbreak in China: Summary of a Report of 72 314 Cases From the Chinese Center for Disease Control and Prevention**. *Jama* 2020.
41. Lu X, Zhang L, Du H, Zhang J, Li YY, Qu J, Zhang W, Wang Y, Bao S, Li Y *et al*: **SARS-CoV-2 Infection in Children**. *The New England journal of medicine* 2020.
42. Zhang R, Wang X, Ni L, Di X, Ma B, Niu S, Liu C, Reiter RJ: **COVID-19: Melatonin as a potential adjuvant treatment**. *Life Sciences* 2020:117583.
43. Reiter RJ, Mayo JC, Tan D-X, Sainz RM, Alatorre-Jimenez M, Qin L: **Melatonin as an antioxidant: under promises but over delivers**. *Journal of pineal research* 2016, **61**(3):253-278.
44. Boga JA, Coto-Montes A, Rosales-Corral SA, Tan DX, Reiter RJ: **Beneficial actions of melatonin in the management of viral infections: a new use for this "molecular handyman"?** *Reviews in medical virology* 2012, **22**(5):323-338.
45. Srinivasan V, Mohamed M, Kato H: **Melatonin in bacterial and viral infections with focus on sepsis: a review**. *Recent patents on endocrine, metabolic & immune drug discovery* 2012, **6**(1):30-39.

46. Reiter RJ, Ma Q, Sharma R: **Treatment of ebola and other infectious diseases: melatonin “goes viral”**. *Melatonin Research* 2020, **3**(1):43-57.

47. Zhou Y, Hou Y, Shen J, Huang Y, Martin W, Cheng F: **Network-based drug repurposing for novel coronavirus 2019-nCoV/SARS-CoV-2**. *Cell discovery* 2020, **6**:14.

48. Acuna-Castroviejo D, Escames G, Venegas C, Diaz-Casado ME, Lima-Cabello E, Lopez LC, Rosales-Corral S, Tan DX, Reiter RJ: **Extrapineal melatonin: sources, regulation, and potential functions**. *Cellular and molecular life sciences : CMLS* 2014, **71**(16):2997-3025.

49. Gitto E, Romeo C, Reiter RJ, Impellizzeri P, Pesce S, Basile M, Antonuccio P, Trimarchi G, Gentile C, Barberi I *et al*: **Melatonin reduces oxidative stress in surgical neonates**. *Journal of pediatric surgery* 2004, **39**(2):184-189; discussion 184-189.

50. Lopez LC, Escames G, Tapias V, Utrilla P, Leon J, Acuna-Castroviejo D: **Identification of an inducible nitric oxide synthase in diaphragm mitochondria from septic mice: its relation with mitochondrial dysfunction and prevention by melatonin**. *The international journal of biochemistry & cell biology* 2006, **38**(2):267-278.

51. Garcia JA, Volt H, Venegas C, Doerrier C, Escames G, Lopez LC, Acuna-Castroviejo D: **Disruption of the NF-kappaB/NLRP3 connection by melatonin requires retinoid-related orphan receptor-alpha and blocks the septic response in mice**. *FASEB journal : official publication of the Federation of American Societies for Experimental Biology* 2015, **29**(9):3863-3875.

52. Yang SF, Chen YS, Chien HW, Wang K, Lin CL, Chiou HL, Lee CY, Chen PN, Hsieh YH: **Melatonin attenuates epidermal growth factor-induced cathepsin S expression in ARPE-19 cells: Implications for proliferative vitreoretinopathy**. *Journal of pineal research* 2020, **68**(1):e12615.

53. Acuña-Castroviejo D, Carretero M, Doerrier C, López LC, García-Corzo L, Tresguerres JA, Escames G: **Melatonin protects lung mitochondria from aging**. *Age (Dordrecht, Netherlands)* 2012, **34**(3):681-692.

54. Kucukakin B, Lykkesfeldt J, Nielsen HJ, Reiter RJ, Rosenberg J, Gogenur I: **Utility of melatonin to treat surgical stress after major vascular surgery--a safety study**. *Journal of pineal research* 2008, **44**(4):426-431.

55. Naguib M, Hammond DL, Schmid PG, 3rd, Baker MT, Cutkomp J, Queral L, Smith T: **Pharmacological effects of intravenous melatonin: comparative studies with thiopental and propofol**. *British journal of anaesthesia* 2003, **90**(4):504-507.

56. Venegas C, Garcia JA, Escames G, Ortiz F, Lopez A, Doerrier C, Garcia-Corzo L, Lopez LC, Reiter RJ, Acuna-Castroviejo D: **Extrapineal melatonin: analysis of its subcellular distribution and daily fluctuations**. *Journal of pineal research* 2012, **52**(2):217-227.

57. Seabra ML, Bignotto M, Pinto LR, Jr., Tufik S: **Randomized, double-blind clinical trial, controlled with placebo, of the toxicology of chronic melatonin treatment**. *Journal of pineal research* 2000, **29**(4):193-200.

58. Molina-Carballo A, Munoz-Hoyos A, Reiter RJ, Sanchez-Forte M, Moreno-Madrid F, Rufo-Campos M, Molina-Font JA, Acuna-Castroviejo D: **Utility of high doses of melatonin as adjunctive anticonvulsant therapy in a child with severe myoclonic epilepsy: two years' experience**. *Journal of pineal research* 1997, **23**(2):97-105.

59. Chahbouni M, Escames G, Venegas C, Sevilla B, Garcia JA, Lopez LC, Munoz-Hoyos A, Molina-Carballo A, Acuna-Castroviejo D: **Melatonin treatment normalizes plasma pro-inflammatory cytokines and nitrosative/oxidative stress in patients suffering from Duchenne muscular dystrophy**. *Journal of pineal research* 2010, **48**(3):282-289.

60. Lopez-Gonzalez A, Alvarez-Sanchez N, Lardone PJ, Cruz-Chamorro I, Martinez-Lopez A, Guerrero JM, Reiter RJ, Carrillo-Vico A: **Melatonin treatment improves primary progressive multiple sclerosis: a case report**. *Journal of pineal research* 2015, **58**(2):173-177.

61. Leonardo-Mendonca RC, Martinez-Nicolas A, de Teresa Galvan C, Ocana-Wilhelmi J, Rusanova I, Guerra-Hernandez E, Escames G, Acuna-Castroviejo D: **The benefits of four weeks of melatonin treatment on circadian patterns in resistance-trained athletes**. *Chronobiology international* 2015, **32**(8):1125-1134.

62. Ramos A, Miguez MP, Morgado S, Sanchez-Correa B, Gordillo JJ, Casado JG, Tarazona R, Regodon S: **Melatonin enhances responsiveness to Dichelobacter nodosus vaccine in sheep and increases peripheral blood CD4 T lymphocytes and IgG-expressing B lymphocytes**. *Veterinary immunology and immunopathology* 2018, **206**:1-8.

63. Crespo E, Macias M, Pozo D, Escames G, Martin M, Vives F, Guerrero JM, Acuna-Castroviejo D: **Melatonin inhibits expression of the inducible NO synthase II in liver and lung and prevents endotoxemia in lipopolysaccharide-induced multiple organ dysfunction syndrome in rats**. *FASEB journal : official publication of the Federation of American Societies for Experimental Biology* 1999, **13**(12):1537-1546.

64. Escames G, Leon J, Macias M, Khaldy H, Acuna-Castroviejo D: **Melatonin counteracts lipopolysaccharide-induced expression and activity of mitochondrial nitric oxide synthase in rats**. *FASEB journal : official publication of the Federation of American Societies for Experimental Biology* 2003, **17**(8):932-934.

65. Escames G, Lopez LC, Ortiz F, Ros E, Acuna-Castroviejo D: **Age-dependent lipopolysaccharide-induced iNOS expression and multiorgan failure in rats: effects of melatonin treatment**. *Experimental gerontology* 2006, **41**(11):1165-1173.

66. Escames G, Acuna-Castroviejo D, Lopez LC, Tan DX, Maldonado MD, Sanchez-Hidalgo M, Leon J, Reiter RJ: **Pharmacological utility of melatonin in the treatment of septic shock: experimental and clinical evidence**. *The Journal of pharmacy and pharmacology* 2006, **58**(9):1153-1165.

67. Acuna-Fernandez C, Marin JS, Diaz-Casado ME, Rusanova I, Darias-Delbey B, Perez-Guillama L, Florido-Ruiz J, Acuna-Castroviejo D: **Daily Changes in The Expression of Clock Genes in Sepsis and Their Relation with Sepsis Outcome and Urinary Excretion of 6-Sulfatoximelatonin**. *Shock (Augusta, Ga)* 2019.

68. Acuna-Castroviejo D, Rahim I, Acuna-Fernandez C, Fernandez-Ortiz M, Solera-Marin J, Sayed RKA, Diaz-Casado ME, Rusanova I, Lopez LC, Escames G: **Melatonin, clock genes and mitochondria in sepsis**. *Cellular and molecular life sciences : CMLS* 2017, **74**(21):3965-3987.

69. Volt H, Garcia JA, Doerrier C, Diaz-Casado ME, Guerra-Librero A, Lopez LC, Escames G, Tresguerres JA, Acuna-Castroviejo D: **Same molecule but different expression: aging and sepsis trigger NLRP3 inflammasome activation, a target of melatonin**. *Journal of pineal research* 2016, **60**(2):193-205.

70. Martin M, Macias M, Escames G, Leon J, Acuna-Castroviejo D: **Melatonin but not vitamins C and E maintains glutathione homeostasis in t-butyl hydroperoxide-induced mitochondrial oxidative stress**. *FASEB journal : official publication of the Federation of American Societies for Experimental Biology* 2000, **14**(12):1677-1679.

71. Escames G, Lopez LC, Ortiz F, Lopez A, Garcia JA, Ros E, Acuna-Castroviejo D: **Attenuation of cardiac mitochondrial dysfunction by melatonin in septic mice**. *The FEBS journal* 2007, **274**(8):2135-2147.

72. Rodriguez MI, Escames G, Lopez LC, Garcia JA, Ortiz F, Lopez A, Acuna-Castroviejo D: **Melatonin administration prevents cardiac and diaphragmatic mitochondrial oxidative damage in senescence-accelerated mice**. *The Journal of endocrinology* 2007, **194**(3):637-643.

DocuSign Envelope ID: 07B15123-9340-4064-AFC9-4BCF44F37DA2

15. HOJA DE FIRMAS

A través de su firma, las siguientes partes confirman la aprobación del presente protocolo:

REPRESENTANTE DEL PROMOTOR:

DocuSigned by:

Ramon garcia

FC411220E035451...

Fecha: 07/06/2020

Nombre, título Ramon García Vizcaino

Afiliación CEO PHARMAMEL

INVESTIGADOR PRINCIPAL:

Juan Carlos Figueira Iglesias
Jefe de sección, Medicina Intensiva
Hospital Universitario La Paz

Fecha: **5 de Junio de 2020**

Nombre, título
Afiliación
